# Synthesis and Isolation of Phenol- and Thiol-Derived Epicatechin Adducts Prepared from Avocado Peel Procyanidins Using Centrifugal Partition Chromatography and the Evaluation of Their Antimicrobial and Antioxidant Activity

**DOI:** 10.3390/molecules29122872

**Published:** 2024-06-17

**Authors:** Barbara Berrios-Henríquez, Matías Venegas-Toloza, María Reyes-Fuentes, Felipe Zúñiga-Arbalti, Luis Bustamante, Apolinaria García-Cancino, Julio Alarcón-Enos, Edgar Pastene-Navarrete

**Affiliations:** 1 Department of Microbiology, Faculty of Biological Sciences, Universidad de Concepción, Víctor Lamas 1290, Concepción 4030000, Chile; barbaraberrios@udec.cl (B.B.-H.); mavenegas2017@udec.cl (M.V.-T.); apgarcia@udec.cl (A.G.-C.); 2 Department of Basic Sciences, Faculty of Sciences, Universidad del Bío-Bío, Avenida Andrés Bello 720, Chillán 3800708, Chile; jualarcon@ubiobio.cl; 3 Department of Biochemistry and Molecular Biology, Faculty of Chemical and Pharmaceutical Sciences, Universidad de Chile, Dr. Carlos Lorca Tobar 964, Independencia, Santiago 8380494, Chile; maria.reyes.f@ug.uchile.cl; 4 Department of Clinical Biochemistry and Immunology, Faculty of Pharmacy, Universidad de Concepción, Víctor Lamas 1290, Concepción 4030000, Chile; fzuniga@udec.cl; 5 Department of Instrumental Analysis, Faculty of Pharmacy, Universidad de Concepción, Víctor Lamas 1290, Concepción 4030000, Chile; lbustamante@udec.cl

**Keywords:** centrifugal partition chromatography, DPPH, procyanidins, antimicrobials, biofilms, avocado

## Abstract

Polyphenols from agro-food waste represent a valuable source of bioactive molecules that can be recovered to be used for their functional properties. Another option is to use them as starting material to generate molecules with new and better properties through semi-synthesis. A proanthocyanidin-rich (PACs) extract from avocado peels was used to prepare several semi-synthetic derivatives of epicatechin by acid cleavage in the presence of phenol and thiol nucleophiles. The adducts formed by this reaction were successfully purified using one-step centrifugal partition chromatography (CPC) and identified by chromatographic and spectroscopic methods. The nine derivatives showed a concentration-dependent free radical scavenging activity in the DPPH assay. All compounds were also tested against a panel of pathogenic bacterial strains formed by *Listeria monocytogenes* (ATCC 7644 and 19115), *Staphylococcus aureus* (ATCC 9144), *Escherichia coli* (ATCC 11775 and 25922), and *Salmonella enterica* (ATCC 13076). In addition, adducts were tested against two no-pathogenic strains, *Limosilactobacillus fermentum* UCO-979C and *Lacticaseibacillus rhamnosus* UCO-25A. Overall, thiol-derived adducts displayed antimicrobial properties and, in some specific cases, inhibited biofilm formation, particularly in *Listeria monocytogenes* (ATCC 7644). Interestingly, phenolic adducts were inactive against all the strains and could not inhibit its biofilm formation. Moreover, depending on the structure, in specific cases, biofilm formation was strongly promoted. These findings contribute to demonstrating that CPC is a powerful tool to isolate new semi-synthetic molecules using avocado peels as starting material for PACc extraction. These compounds represent new lead molecules with antioxidant and antimicrobial activity.

## 1. Introduction

**Figure 1:**
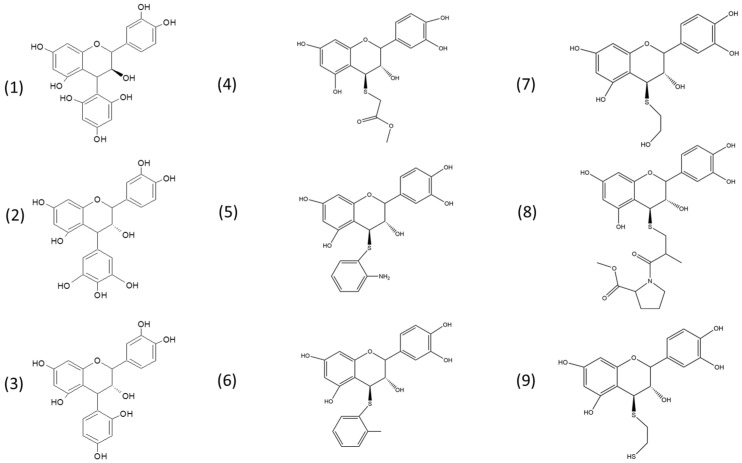
Chemical structures of the phenol and thiol adducts investigated in the present study: epicatechin–phloroglucinol (**1**); epicatechin–pyrogallol (**2**); epicatechin–resorcinol (**3**); epicatechin–methyl-thioglycolate (**4**); epicatechin–2-aminothiophenol (**5**); epicatechin–2-methylthiophenol (**6**); epicatechin–mercaptoethanol (**7**); epicatechin–S-captopril methyl ester (**8**); and epicatechin–1,2-ethanedithiol (**9**).

Proanthocyanidins (PACs) are oligomeric forms of flavan-3-ols-like catechins or gallocatechins. The peel of different fruits has important amounts of PACs that can be extracted for use as antioxidants or preservatives [1]. In previous works, we obtained PAC-enriched extracts from *Peumus boldus* leaves, grape wastes, and avocado and apple peels. In these works, antimicrobial and enzyme inhibitor properties were assessed [2,3,4,5]. Such bioactivities are related to several PAC structure features such as molecular size and hydroxylation pattern [5]. In 2015, the United States Department of Agriculture (USDA) updated a database referring to the content of procyanidins in different foods, where the presence of dimers, trimers, oligomers, and polymers was evaluated. This document highlights foods such as cinnamon (*Cinnamun aromaticum*), blueberry (*Vaccinium corymbosum*), grape seeds (*Vitis vinifera*), cocoa seeds (*Tehobroma cacao*), and sorghum (*Sorghum bicolor*), for which the amounts of oligomers and polymers were the highest [6]. In Chile, large amounts of avocado peels are generated as agro-waste [7], and polymeric PACs extracted from these products could be used as starting material for the semi-synthesis of (epi)-catechin-derived compounds. To determine the mean degree of polymerization (mDP), acid-catalyzed cleavage of these PACs allows for the synthesis of flavan-3-ol adducts via nucleophilic attack with phloroglucinol, resorcinol, or pyrogallol, as was demonstrated in previous works [8,9,10]. In strongly acidic conditions, PACs are degraded, releasing extension subunits as electrophilic (epi)-catechin intermediates, which are trapped by nucleophilic agents, yielding characteristic adducts [11]. For instance, various low-molecular derivatives from PACs from diverse sources can be easily synthesized via nucleophilic attack. Our group found that such compounds inhibited *Helicobacter pylori* ATCC 43504 adherence to AGS cells and reduced IL-8 release [12]. Most recently, we reported that epicatechin–pyrogallol, catechin–pyrogallol, and catechin–phloroglucinol adducts inhibit human tau aggregation (a hallmark of Alzheimer’s disease) and significantly increase neuritogenesis in a dose-dependent manner. Among these adducts, phloroglucinol-derived compounds were the most active molecules, suggesting that the introduction of a phloroglucinol group may enhance the neuroprotective activity of the catechin-derived compounds [13]. However, this reaction can be carried out not only with phenols such as phloroglucinol, resorcinol, and pyrogallol but also with other nucleophiles like the thiol compounds cysteine, cysteamine, and toluene-α-thiol. Thus far, only the biological activity of adducts prepared using cysteine, cysteamine, and toluene-α-thiol has been reported regarding its anti-inflammatory, antioxidant, and anticancer activities [14,15]. It should be noted, however, that the antimicrobial activity of phenol and thiol adduct derivatives has been scarcely investigated. Nonetheless, by observing the structure of such compounds, it is evident that they could not work well given their high polarity compared with the superhydrophobic properties observed in biofilm-producing bacteria. These biofilms not only permit capturing nutrients but also avoid mechanical and chemical clearance from different surfaces. In addition, recent experimental evidence suggests that biofilm is a virulence factor in a bacterial community because the bacterial cells residing in the biofilm may acquire new virulence attributes that free-living bacteria do not possess [16]. These features enable an increasing rate of bacterial resistance and tolerance. In fact, the National Institutes of Health (NIH) report that around 80% of chronic bacterial infections observed in humans are caused by biofilm-producing bacteria [17]. The resistance of these “superbugs” could arise against a vast group of commonly used antibiotics, like methicillin-resistant *Staphylococcus aureus* (MRSA), vancomycin-resistant *Enterococcus* (VRE), and multidrug-resistant *Mycobacterium tuberculosis* (MDR-TB) [18]. Among the most relevant infections, we can mention: burn wound infections, ear infections, catheter infections, endocarditis, etc. Taking into account the state-of-the-art, three different strategies could be used to reduce biofilms: (1) blocking bacterial adhesion to surface (solid or cells); (2) interrupting biofilm growth and its architecture with the aim to increase antibiotic permeability, and (3) avoiding biofilm maturation and/or promoting dispersion and degradation [19]. In most of the above scenarios, to reach their molecular targets, the semisynthetic adducts must have sufficient polarity to cross the bacterial lipid membrane. So, we believe that their penetration must be improved by making them more lipophilic. Thereby, in the present work, we will increase the lipophilic character of the scaffolds (flavan-3-ol adducts) using lipophilic thiol nucleophiles (Figure 1), as inspired by early works [20].

Purification of PAC adducts can be quite challenging given the complexity of the phenolic extracts. Regarding the separation strategies of the different adducts obtained for procyanidins, initially, these mainly focused on the derivatives obtained with phenolic nucleophiles [10]. Thus, their purification has been approached by preparative HPLC [21] using a gradient of water (A) and acetonitrile 0.5% acetic acid (B), allowing for the compounds to be obtained on a milligram scale. On the other hand, the use of high-speed counter-current chromatography (HSCCC) is suitable for the isolation of greater quantities of the same compounds. In contrast to (HPLC), where columns filled with solid stationary phases are used, counter-current separation (CCS), uses a mixture of solvents (normally hexane, ethyl acetate, methanol, and water), which generate two immiscible phases with each other, corresponding, in this case, to the mobile and stationary phases. The chosen liquid stationary phase is placed in the rotor of the equipment and by means of a centrifugal force is retained inside the partition cells. Afterward, the mobile phase is pumped inside the rotor and, in this way, a phenomenon of emulsification and continuous separation of the phases occurs, also allowing for an increase in the interfacial area for mass transfer. The components ready for fractionation achieve their separation according to their partition coefficient (KD), the latter representing the relationship between the concentration of the components in both phases [22]. Typically, the solvent systems used for HSCCC purification of phenolic adducts were hexane–ethyl acetate–methanol–water (0.1:5:0.1:5, *v*/*v*/*v*/*v*) and (1.5:10:1.5:10, *v*/*v*/*v*/*v*) in the “head to tail” or descending mode [23]. In other studies, separations were performed by alternating descending and ascending modes using hexane–ethyl acetate–water (1:80:80, *v*/*v*/*v*) [24]. These compounds were also separated in a single step by centrifugal partition chromatography (CPC) with a two-phase solvent system composed of hexane–ethyl acetate–methanol–water (1:9:1:9 *v*/*v*/*v*/*v*) in ascending mode [12]. In the case of adducts obtained with thiol nucleophiles, the choice of the separation method is linked to the polarity of the adduct obtained. Some authors [25,26,27,28] reported the use of cysteamine as a nucleophilic agent and addressed purification using a combination of adsorption chromatography in Amberlite XAD-16, cation exchange, and reverse phase semipreparative HPLC. For instance, Selga and coworkers obtained 263 g of the cysteamine–epicatechin adduct with a purity of 35% by reversed-phase high-performance liquid chromatography starting from 17 kg of pine bark (*Pinus pinaster*) in a process that took around 10 h [29]. The same group obtained 17 g of 63% pure cysteamine–epicatechin (acetate salt) adduct from 35 kg of grape pomace [26]. Several catechin and tiopronin derivatives were purified by HSCCC after depolymerization of grape seed [30]. The solvent system used by this group was n-hexane–ethyl acetate–methanol–water (0.12:1.5:0.5:1 *v*/*v*/*v*/*v*) in descending mode. After the introduction of 400 mg of the tiopronin degradation products mixture and 300 min of elution, three new impure compounds were obtained, which required an additional purification step by semipreparative HPLC. These compounds showed antimicrobial effects on *Staphylococcus aureus* and *Escherichia coli*. The use of the antihypertensive drug captopril as a nucleophilic agent for the degradation of procyanidins has also been recently reported. In a study by Cui and colleagues [24], given the lower polarity of this adduct, they were able to purify it by normal column chromatography using silica gel and elution with dichloromethane/ethanol (15:1 *v*/*v*). In a recent work by Tian and collaborators [31], using HSCCC, it was possible to purify eight new thiol adducts of captopril, L-cysteine, and tiopronin after the degradation reaction of grape seed extracts. The authors reported that different solvent systems were necessary for the isolation of thiol adducts in their study. So, n-hexane–ethyl acetate–methanol–water systems were used for the most lipophilic compounds, while n-butanol–ethyl acetate–methanol–water systems were necessary to purify most polar adducts.

The purpose of the present study was to obtain a wide range of epicatechin adducts from avocado PACs as starting material using phenol and thiol nucleophiles with different polarities and explore the advantage of centrifugal partition chromatography (CPC) for the fast one-step isolation of these compounds. This versatile liquid–liquid separation methodology belongs to the counter-current separation (CCS) techniques that allowed for the purification of target compounds with diverse polarity in short times [32]. Compared with HSCCC and HPLC, CPC has a large loading capacity due to its high retention of the stationary phase and a greater theoretical plate height [33,34]. Finally, after purification by CPC, we compared their antioxidant and antimicrobial activity over pathogenic and non-pathogenic strains.

## 2. Results and Discussion

### 2.1. Extraction of Avocado Peel Procyanidins and Semi-Synthesis of Phenol- and Thiol-Derivatives

**Figure 2:**
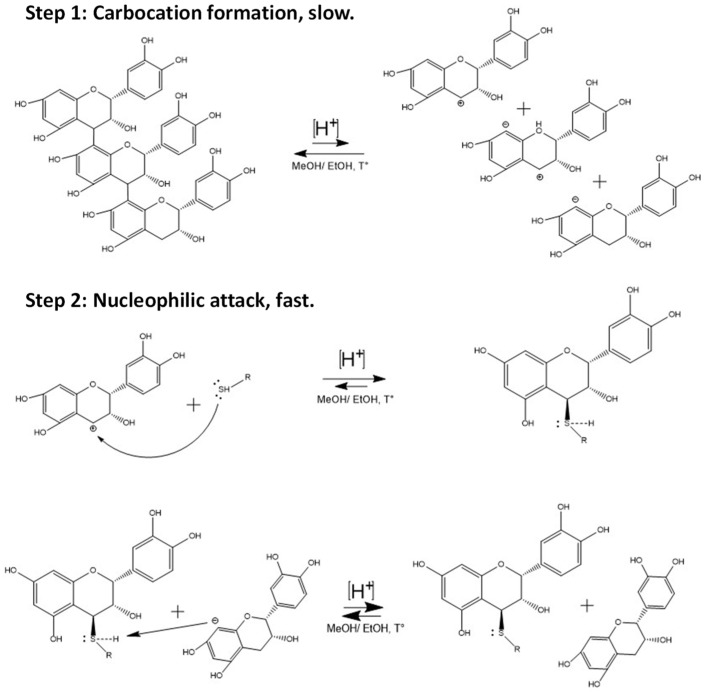
General scheme of epicatechin thiol conjugates synthesis by depolymerization of avocado peel proanthocyanidins.

For the hemisynthesis of the phenol and thiol adducts, a depolymerization reaction of type B proanthocyanidins from avocado peel was carried out in an acidic medium. As depicted in the general scheme of the reaction (Figure 2), the reaction mechanism corresponds to a type 1 nucleophilic substitution reaction (SN1). In such conditions, depolymerization occurs thanks to the acidic environment supplied by the 37% fuming HCL that gives rise to the formation of the carbocation [9,34,35,36]. The formation of these last structures is the point of the highest energy and is considered the limiting step, which determines the reaction rate. Other factors to consider regarding the formation of the carbocation are the characteristics of the leaving group, such as its degree of acidity and stability, in this case, the negative charge generated in the A ring of (−)-epicatechin is stabilized by resonance [11]. On the other hand, the use of methanol and ethanol as protic media is essential to stabilize the carbocation through solvation. However, depending on the strength of the nucleophile, this type of media can interfere with the reaction. In the specific case of this hemisynthesis, the thiol nucleophiles had greater nucleophilic strength than the medium, so the structures formed did not deviate from what was expected.

### 2.2. Isolation of Epicatechin Phenol and Thiol Adducts by Preparative Centrifugal Partition Chromatography (CPC)

To separate the reaction products, the preparative CPC methodology was used. After the extraction of PACs, we evaluated the suitability of CPC for the isolation of adducts using HPLC analysis (λ = 280 nm) of upper and lower phases performed at an analytical scale (Table 1). This pre-analysis of partition coefficients using the shake flask method allowed us to select the best biphasic solvent systems. The peak area of the target compound in the upper and lower phases via HPLC-UV analysis enables the determination of the specific KD values. Once the KD values were calculated for this nine-target compound, the semi-preparative fractionation of 1 g of depolymerized avocado PACs was carried by CPC (10 mL injection loop). Afterward, two phenolic adducts (phloroglucinol and pyrogallol) were purified by CPC in ascending mode using solvent system C (1:9:1:9 *v*/*v*/*v*/*v*) from HEMWat series (hexane-EtOAc-MeOH-H_2_O), following similar conditions published in our previous work [12]. The stationary phase retention values for this system were 84%. In the case of the resorcinol adduct, solvent K showed better results because, in system C, a larger retention time was necessary to obtain this compound in ascending mode. The stationary phase retention values for the K system were 82%.

The analysis by liquid chromatography–mass spectrometry (LC-ESI-MS) in negative ion mode for (**1**–**2**) gave [M − H]^−^ ions at *m*/*z* 413.5. Both nucleophiles were used in the routine analysis of the mean degree of polymerization (mDP) of proanthocyanidins and could be easily identified by their retention times in the HPLC-UV analysis. As expected, epicatechin–resorcinol (**3**) exhibits a [M − H]^−^ ion at *m*/*z* 398.9. These results agree with previous works [9,10,12].

**Figure 3:**
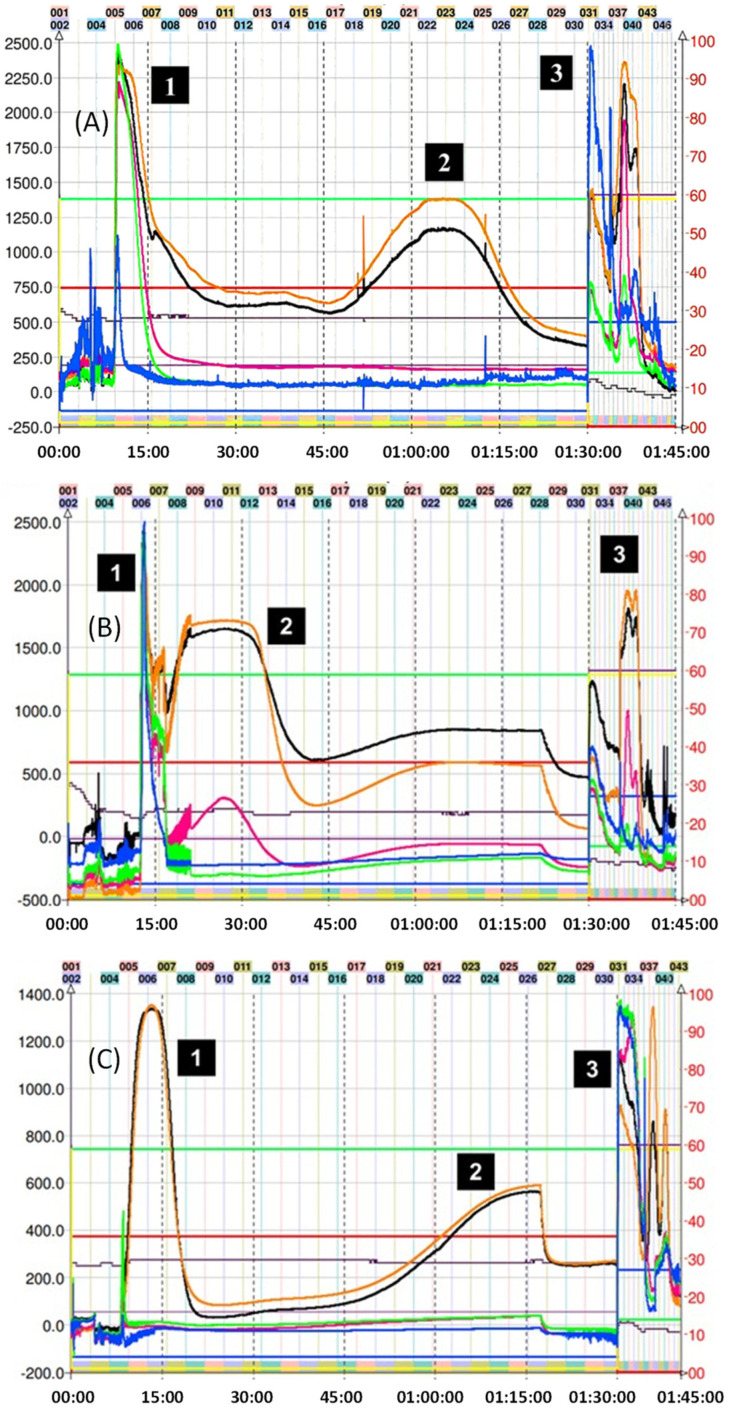
Chromatogram of methyl thioglycolate, 2–aminothiophenol–, and 2–methyl thiophenol–induced depolymerization mix of avocado peel PAC extract in ascending mode with solvent system K. (**A**) (1) Excess of methyl thioglycolate, (2) methyl thioglycolate adduct (**4**), and (3) final extrusion process and output of the highly polar compounds from the avocado peel PAC extract. (**B**) (1) Excess of 2–aminothiophenol, (2) 2–amino thiophenol adduct (**5**), and (3) final extrusion process and output of the highly polar compounds from the avocado peel PAC extract. (**C**) (1) 2–methyl thiophenol adduct (**6**), (2) epicatechin, and (3) final extrusion process and output of the highly polar compounds from the avocado peel PAC extract (unreacted 2–aminothiophenol is seen as a small peak at 7.5 min). Black trace corresponds to scanning mode 200–600 nm; orange line is the register at 280 nm; violet line is the register at 330 nm; green line is the register at 360 nm; and blue line corresponds to the register at 430 nm.

For the CPC isolation of most thiol adducts, system K not only allowed for the neat separation of the compounds but also produced fractionation in a reduced time since only a single run was necessary. Figure 3 shows the chromatogram obtained in the fractionation of the thiol adducts (**4**–**6**), where system K of the Arizona biphasic system was used in ascending mode (Nonpolar phase: mobile phase, polar phase: stationary phase). In this figure, it is possible to observe adduct peaks within the first 90 min of the run. The excess nucleophilic reagent in all cases elutes at the beginning. Subsequently, thanks to the increase in lipophilic character (compared with the epicatechin monomer), the peaks of the (**4**–**6**) adducts were collected and analyzed by TLC and HPLC.

**Figure 4:**
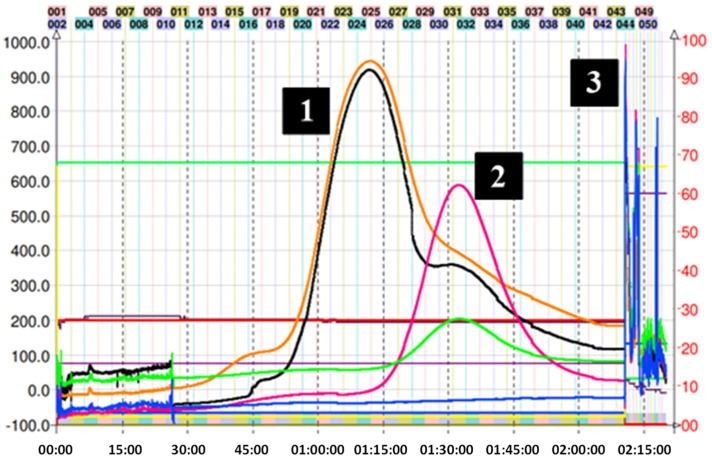
Chromatogram of mercaptoethanol–induced depolymerization mix of avocado peel PACs in ascending mode with solvent system H. (1) Mercaptoethanol adduct (**7**), (2) excess of mercaptoethanol, and (3) final extrusion process and output of the highly polar compounds from the avocado peel PAC extract. Black trace corresponds to scanning mode 200–600 nm; orange line is the register at 280 nm; violet line is the register at 330 nm; green line is the register at 360 nm; and blue line corresponds to the register at 430 nm.

On the other hand, for adduct (**7**), given the expected structure, and corroborating its average octanol/water partition coefficient (Ko/w = 0.7) using the in silico pharmacokinetic and toxicity algorithm SwissADME, it was estimated that the solvent mixture necessary for a correct separation should be more polar. So, the decision was made to go down a step in the Arizona solvent system, finally using the H system (1:3:1:3 *v*/*v*/*v*/*v*). Figure 4 shows the chromatogram corresponding to the separation of adduct (**7**). In the latter, a slight overlap of the peaks can be observed between 45 and 120 min of the run. However, despite the low resolution obtained in this fractionation, collecting samples in small-volume tubes allows for manual cuts, thus delimiting the presence of other components in the sample. In this particular case, the sample collection was carried out by gathering the content present between tubes 18 and 23 (55–72 min). The overlap of the peaks in Figure 4 can be explained by the similar KD for mercaptoethanol and its epicatechin derivative. Ito et al. mentioned that compounds with an upper/lower partition coefficient (KD up/low) between 0.5 and 1.0 (based on the solvent system used) present better fractionation results when the descending mode is used [22]. Despite these data, the HPLC determination of KD up/low for (**7**) in system H was 2.3. Even with the longer elution time, there was still some overlap; therefore, we decided to use ascending mode since it is easier to obtain the product from the organic solvent by simple distillation. The sections of the peak of adduct (**7**) that appear superimposed were pooled and reinjected to recover the remaining compound.

**Figure 5:**
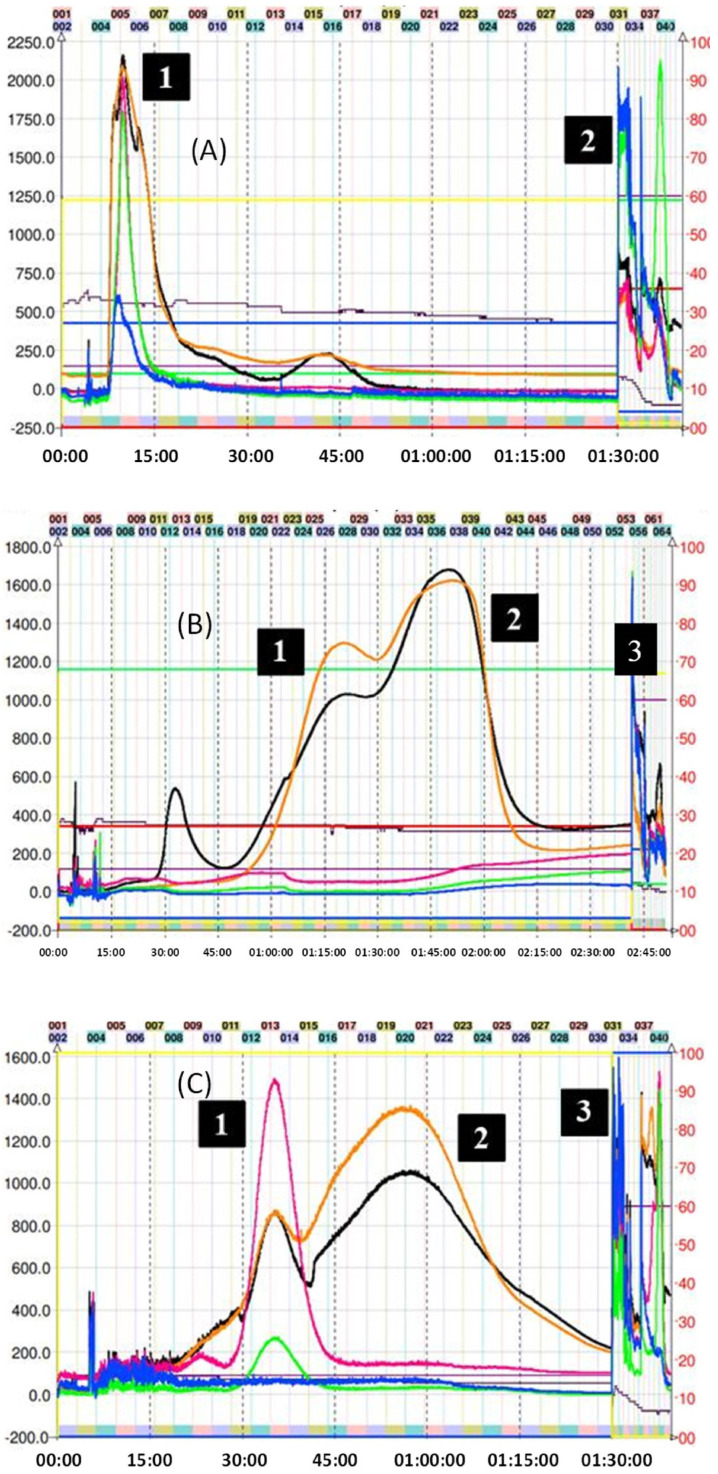
(**A**) Chromatogram of captopril-induced depolymerization mix of avocado peel PAC extract in ascending mode with solvent system K. (1) Unresolved captopril adduct (**8**) and (2) final extrusion process with excess captopril and output of the highly polar compounds from the avocado peel PACs extract. (**B**) Chromatogram of captopril–induced depolymerization mix of avocado peel PAC extract in ascending mode with solvent system H. (1) Impurities, (2) unresolved captopril adduct (**8**), and (3) final extrusion process and output of the highly polar compounds from the avocado peel PAC extract. (**C**) CPC trace of captopril adduct (**8**) using the optimized system: hexane-EtOAc–MeOH–H_2_O 0.05:1.5:0.5:1.2 *v*/*v*/*v*/*v* in descending mode, (1) excess captopril and avocado peel PAC extract (2) captopril adduct (**8**), and (3) final extrusion process and output of the most lipophilic compounds from the avocado peel PAC extract. Black trace corresponds to scanning mode 200-600 nm; orange line is the register at 280 nm; violet line is the register at 330 nm; green line is the register at 360 nm; and blue line corresponds to the register at 430 nm.

Purification of the captopril adduct (**8**) turned out to be the most laborious and required an exploration of solvents in proportions different from those found in the Arizona tables. In the first chromatographic run, which corresponds to Figure 5A, system K was used in descending mode. This is because the presence of carbonyl and alcohol groups belonging to the captopril molecule would theoretically increase the polarity of the molecule when compared with the addition of other groups such as 2-methylthiophenol. Consequently, the use of descending or the “head to tail” mode (similar to a reverse phase in HPLC) would preliminarily allow for the output of more polar compounds. As can be seen in Figure 5A, this phenomenon occurs as expected. However, the separation of the various reaction components with respect to compound (**8**) was not optimal, and a completely overlapping peak was observed. Therefore, we decide to use a more polar two-phase system (H system = 1:3:1:3 *v*/*v*/*v*/*v*) in ascending mode. The chromatogram of this second attempt at purification can be seen in Figure 5B. In this case, it is possible to observe the presence of two peaks with different intensities, the second corresponding to compound (**8**). Unfortunately, the resolution provided by this method is not sufficient to perform the separation in a single step. Although it is possible to collect the tubes (34 to 42) that contain the adduct, its presence between the previous tubes would require a second CPC run, increasing operating expenses and time invested. In this separation, KD of (**8**) was 4.40. Finally, compound (**8**) was subsequently purified following the strategies suggested in the work published by Tian and coworkers [31]. In the latter, the authors optimize the separation of the reaction components of the captopril adduct. Hence, they used five biphasic systems based on hexane, ethyl acetate, methanol, and water, determining, in turn, the values of the KD and the separation factor α of the following solutes: (+)–catechin, (−)–epicatechin, (−)–epicatechin–3–O–gallate, (−)–epicatechin–4β–captopril methyl ester, and (−)–epicatechin–3–O–gallate–4β–captopril methyl ester. These systems are not described in the proportions illustrated in the Arizona tables, although they are derived from them. Finally, it was determined that the best solvent system for the purification of the adduct results from the ratio 0.05:1.5:0.5:1.2 of hexane–EtOAc–MeOH–H_2_O, respectively [31]. In this separation, the KD of (**8**) was 1.92. Figure 5C illustrates the results of this last purification. The use of the previously mentioned system allowed for increasing the resolution of the adduct concerning the other reaction components, allowing for its correct separation, and locating the adduct from tubes 16 to 30. CPC was used in ascending mode. Therefore, the first peak observed corresponds to the excess of the captopril molecule. After 90 min of running, the extrusion process begins, where the output of the other components of the avocado PAC extracts that do not participate in the reaction is observed.

**Figure 6:**
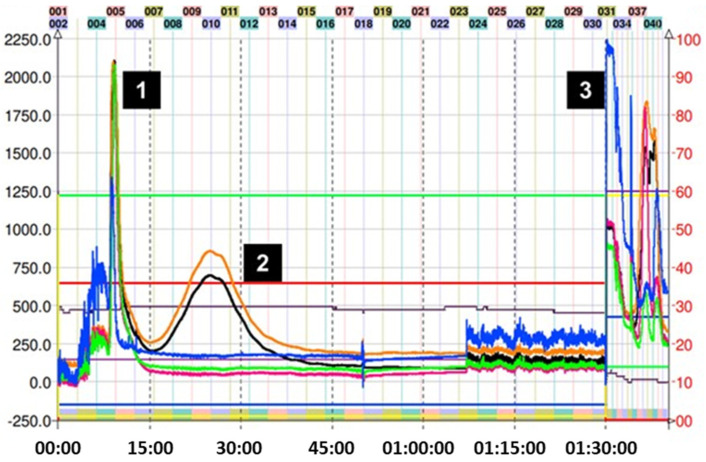
Chromatogram of ethane dithiol–induced depolymerization mix of avocado peel PAC extract in ascending mode with solvent system K. (1) Excess ethane dithiol, (2) ethane dithiol adduct (**9**), and (3) final extrusion process and output of the highly polar compounds from the avocado peel PAC extract. Black trace corresponds to scanning mode 200–600 nm; orange line is the register at 280 nm; violet line is the register at 330 nm; green line is the register at 360 nm; and blue line corresponds to the register at 430 nm.

As seen in Figure 6, the purification of adduct (**9**) was achieved in one step with system K, which successfully obtained the product in a few tubes. Since the preparation of this adduct was the one that obtained the lowest yield, the direct isolation using CPC was important to reduce additional losses.

The yields of the hemisyntheses after CPC purification are shown in Table 2. In the latter, it is observed that the yields in the formation of the adducts range between 14 and 22% based on the total extract used. These yield values are closely related to the presence of PAC polymers and oligomers present in the avocado extract. Using larger amounts of polymer, the reaction is favored by the increased availability of electrophilic sites [37]. Therefore, using other sources of procyanidins could be useful to increase the reaction yields. However, the degree of polymerization present in these foods is not the unique parameter to consider for improving reaction yields since the C4/C8 linkage that gives rise to the formation of B-type procyanidins is also important. Nevertheless, in several of these foods, we not only find B-type procyanidins but also A-type procyanidins with an ether bond at C_2_-O-C_7_. The evidence indicates that acid depolymerization or acidolysis is effective only in type B procyanidins, so foods such as cinnamon, which rich are in A-type procyanidins, would not be as useful in that case [38,39,40]. Considering these data, it is possible to evaluate the use of various foods as rich sources of procyanidins. It is important to highlight that the use of avocado peel to obtain these resources gives it a new value as a raw material, thus contributing to the circular economy and the maximum use of natural products.

### 2.3. HPLC-ESI-QTOF-MS-MS Analysis of Thiol Adducts

As indicated above, the phenolic adducts (**1**–**3**) were prepared and purified according to a previously published protocol. Similarly, the chromatographic analysis and the structures of said derivatives fully corresponded to the reported data [12]. The characteristic fragmentation pathway of proanthocyanidins was analyzed based on previously published works [41,42,43]. The other six adducts were identified by HPLC–ESI–QTOF–MS-MS, and their spectra and structure skeletons are shown in Figures S1–S6. The accurate mass measurements, retention time, formulae, and errors for all compounds are summarized in Table S1 as well as the main product ions observed in the MS–MS spectrum. In Figure S1 (Supplementary Materials, Figure S1), it is possible to observe the presence of adduct (**4**) with an *m*/*z* at 393.06, which corresponds to the negatively charged pseudomolecular ion [M − H]^−^. A fragment at *m*/*z* 287.05 corresponds to the monomeric ion epicatechin after the loss of 106 amu, corresponding to –HSCH_2_COOCH_3_ (methyl thioglycolate). In the same figure, it is possible to observe the appearance of characteristic ions at *m*/*z* 787.14 and 1181.22, which could be explained by the coupling of two or three base adducts (**4**), which can be corroborated in the MS2 spectra. On the other hand, a fragment at *m*/*z* of 681.13 can be observed in MS1, this being the result of the partial depolymerization of a B-type procyanidin trimer, with the consequent substitution with methyl thioglycolate. This reaction results in the formation of a dimer of adduct (**4**). The MS2 spectrum of this compound shows an ion fragment at *m*/*z* 287.06, corresponding to the monomeric ion epicatechin. In the mass spectrum of adduct (**5**) (Supplementary Materials, Figure S2), the presence of the pseudomolecular ion at *m*/*z* = 412.09 is observed. The structure with code [2M − H]^−^ (*m*/*z* = 825.18) corresponds to the adduct [2M − H]^−^ formed during the MS analysis. This occurs through the association of two base adducts (**5**). A fragment at *m*/*z* 287.05 corresponds to the monomeric ion epicatechin after the loss of 125 amu, corresponding to –C_6_H_4_(SH)(NH_2_), (2-aminothiophenol). Furthermore, it is possible to observe the presence of the (**5**) dimer with *m*/*z* = 700.15 in MS1. These procyanidin B2 adducts derive from larger structures that were not able to depolymerize completely during the nucleophilic attack reaction. As expected, after the loss of 125 amu, the MS2 spectrum showed a characteristic ion fragment at 287.0527, corresponding to the monomeric epicatechin. In MS1 for adduct (**6**) (Supplementary Materials, Figure S3), the pseudomolecular ion [M − H]^−^ at *m*/*z* = 411.09 is observed, as well as the adduct [2M − H]^−^ and derived from the association between two base adducts of (**6**) (*m*/*z* = 823.19). A low-intensity signal for the [3M − H]^−^ specimen derived from the association between three base adducts of (**6**) is also observed. The MS1 spectrum for the dimer of (**6**) with *m*/*z* = 699.15 is also presented in the lower panel of Figure S3. As expected, after the loss of 124 amu, the MS2 spectrum showed a characteristic ion fragment at *m*/*z* 287.05, corresponding to the monomeric epicatechin. In Figures S3 and S4, signals at *m*/*z* = 575.10 are observed for adducts (**5**) and (**6**). These fragments are not related to the adducts and probably correspond to the trace amount of A-type procyanidins recalcitrant to the depolymerization reaction. On the other hand, some studies describe the transformation of B-type procyanidins into A-type procyanidins through oxidation reactions. For this transformation to occur, the hydride ion must be released from C2 in ring C, so that subsequently, the oxidation of a hydroxyl group occurs in ring B, giving way to the quinone. Finally, the oxygen in the hydroxyl group at C_7_ attacks C_2_, deprotonating this intermediary and generating an additional C_2_-O-C_7_ bond [44,45]. Figure S4 shows the mass spectrum of adduct (**7**). In the latter, the appearance of the expected pseudomolecular ion at *m*/*z* = 365.07, corresponding to the 2-mercaptoethanol adduct, is evident. After the loss of 78 amu, corresponding to –HSCH_2_CH_2_OH_2_ (mercaptoethanol), the ion fragment at *m*/*z* 287.06 appears. The dimeric structure of the adduct is observed at *m*/*z* = 653.13. On the other hand, it is possible to appreciate the signals of the adducts [2M − H]^−^, [3M − H]^−^, and [4M − H]^−^ formed within the source due to the association between two or more (**7**) adducts.

The mass spectrum of adduct (**8**) is presented in S5 (Supplementary Materials, Figure S5). The pseudomolecular ion [M − H]^−^ at *m*/*z* = 518.15 is observed. The dimeric derivative is observed at *m*/*z* = 806.21. Contrary to adduct (**7**), which can form adducts derived from the association between four base adducts, adduct (**8**) only forms adducts derived from the union of two base adducts. This signal is shown with the code [2M − H]^−^. The mass spectrum of adduct (**9**) is shown in Figure S6 (Supplementary Materials, Figure S6). The pseudomolecular ion [M − H]^−^ corresponding to the adduct gave a signal at *m*/*z* = 381.05. The presence of dimeric adduct (**9**) derived from the B-type procyanidin plus 1,2–ethanedithiol with *m*/*z* value = 669.11 is observed. The *m*/*z* value suggests that the probable structure of this compound could correspond to two epicatechin units connected by 1,2–ethanedithiol rather than a dimeric procyanidin with a 1,2–ethanedithiol group attached at C-4. There are no previous reports of this type of structure, and, in the future efforts, will be made to purify it and unambiguously establish its structure. In a similar way to the previous spectra, the formation of dimers and trimers within the source is evident, generating signals [2M − H]^−^ at *m*/*z* = 763.10 and [3M − H]^−^ at *m*/*z* 1145.163, respectively.

### 2.4. Antioxidant Activity

**Figure 7:**
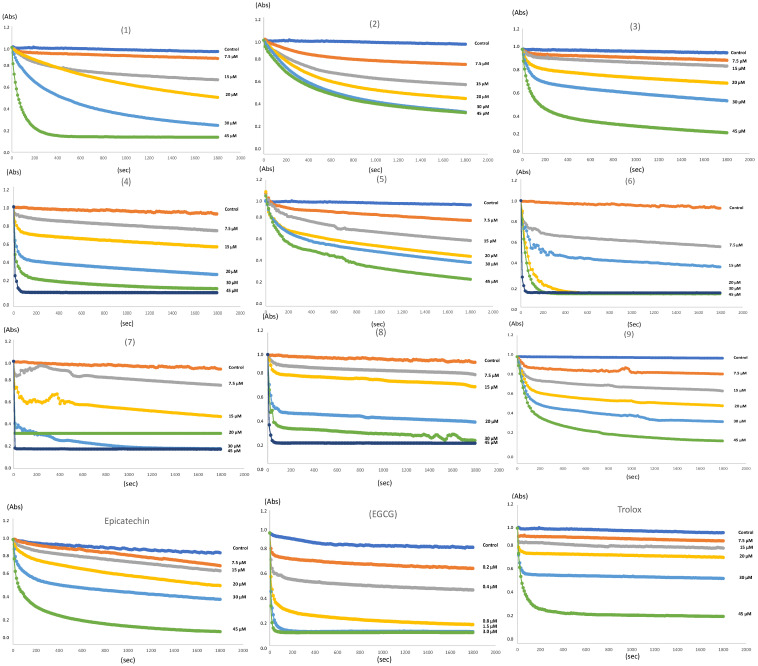
Kinetics curves of DPPH scavenging activity of adducts (**1**–**9**). All adducts and Trolox samples were prepared at 7.5–45 μM. Epigallocatechin gallate (EGCG) was prepared at 0.2–3 μM.

The radical scavenging activity of all adducts was evaluated using a DPPH (2,2–diphenyl–1–picrylhydrazyl) radical (DPPH^•^) assay. This antioxidant test is based on the reduction of the purple stable DPPH^•^ by hydrogen atom or electron transfer reactions in the presence of antioxidant molecules. The latter promotes the decolorization of the purple DPPH radical into a pale-yellow hydrazine compound (DPPH_2_) [46]. The results of the DPPH assay are presented in Table 3. It can be concluded that, in general, the thiol derivatives of epicatechin have a slightly higher antioxidant capacity than the three phenolic adducts derived from pyrogallol (**1**), phloroglucinol (**2**), and resorcinol (**3**). In particular, the effect of adducts (**6**) and (**7**) stand out, which even have a lower IC_50_ value than Trolox. In Figure 7, it is evidenced that all adducts show concentration-dependent radical scavenging ability. Interestingly, although the decolorization kinetics of the DPPH radical are similar among the phenolic derivatives, thiol adducts, such as (**4**) and (**6**–**8**), present a different behavior. The rapid discoloration phase for these adducts is similar to that observed for Trolox (Figure 7). The above suggests a fast interaction with the DPPH radicals. In the recent work by Angeli and collaborators [47], the authors described that flavan–3–ols (catechin, epicatechin), flavonoids (quercetin, rutin), and tannins (tannic acid, ellagic acid), presented a predominating side reaction (k_2_ from 15 to 60 M^−1^ s^−1^) compared with Trolox, α-tocopherol, and ascorbic acid. Using the stop-flow approach, in these latter compounds, a stoichiometric factor value of 2 was calculated, whereas flavonoids and catechins had stoichiometric factor values of 4.8 and 5.8, respectively. Furthermore, the first-order constants for this type of compound bordered on values of k_1_ = 21,100 ± 570 M^−1^ s^−1^. Typically, these types of reactions have also been reported with antioxidants having carboxylic functions [48]. So, the fast kinetic behavior observed for captopril adduct (**8**) and methyl thioglycolate adduct (**4**) suggests that these compounds are antioxidants with low capacity (low stoichiometry factor “n”) but high activity (high value of k_1_). It is intriguing that even though adducts (**4**) and (**8**) are epicatechin derivatives, their behavior is much closer to antioxidants such as Trolox or ascorbic acid, suggesting that the introduction of substituents that include the carboxyl or ester function has a strong impact on the activity and antioxidant capacity. It is well-known that captopril has antioxidant activity upon DPPH, ABTS, and galvynoxil radicals since it is able to donate hydrogen from –SH to DPPH [49,50]. The same mechanism has been reported for thiophenols and aliphatic thiols [51]. Nevertheless, in adducts (**4**) and (**8**), the S atom of this group forms a bond with C-4 and, therefore, no longer has the H atom to transfer to DPPH. Even more striking is the fact that ethane-1,2-dithiol adduct (**9**), despite having a free –SH group, has a behavior very similar to flavan-3-ols such as epicatechin (Figure 7).

### 2.5. Antimicrobial and Anti-Biofilm Properties

The effectiveness of the compounds was evaluated using various tests on Gram-positive and -negative strains. Bacterial susceptibility tests were carried out in agar plates and using a final concentration of 500 μg of the adduct per well or disk. Additionally, the ability to inhibit biofilm formation was evaluated in 96-well microplates, using crystal violet and rezasurin to assess biofilm formation and cell viability, respectively. According to the results presented in Table 4, the semisynthetic adducts derived from epicatechin (**1**–**3**) did not show antibacterial activity at the concentration of 500 µg/mL against all the bacterial strains tested. Regarding the control antibiotics, the strains *L. fermentum* UCO 979C, *L. rhamnosus* UCO 25A, and *L. monocytogenes* 19115 were sensitive to amoxicillin (AMX) and metronidazole (MTZ), while *E. coli* 25922 was resistant to both antimicrobials tested.

In general, when observing the data in Table 5, it can be confirmed that thiol-derived adducts **4**–**9** have a more selective activity on pathogenic bacteria and do not affect the two probiotic strains used as a reference. Specifically, adducts (**4**–**6**) showed activity on all pathogenic strains, while (**7**) was not active upon *S. aureus* 9144 and (**8**) did not affect *L. monocytogenes* strains. In the same way, it can be said that the activity on *E. coli* and *S. enterica* strains was moderate for all the adducts tested. Among the adducts with activity against *L. monocytogenes* strains, compound (**5**) stands out with inhibition zones of around 30 mm, closer to the inhibition zone of gentamicin (Figure S7). Adduct (**5**) is the epicatechin–2–aminothiophenol and is the only molecule harboring an amino group. In agreement with our findings, recently, it was demonstrated that the introduction of amino-thiophenol as a linker in leuromutilin, a tricyclic diterpenoid natural product, allowed for obtaining a new type of antibiotic with a powerful in vitro and in vivo effect against *S. aureus* MSRA and *E. coli* (ATCC 25922) [52,53]. Therefore, these adducts could be used in the future as a new scaffold directed against this pathogen. Among the thiol group of adducts, epicatechin–mercaptoethanol (**7**) was the weaker compound with inhibition zones ranging from 7 to 12 mm. Interestingly, this compound is also the most polar among thiol-derived epicatechin groups (Figure S8, HPLC).

**Figure 8:**
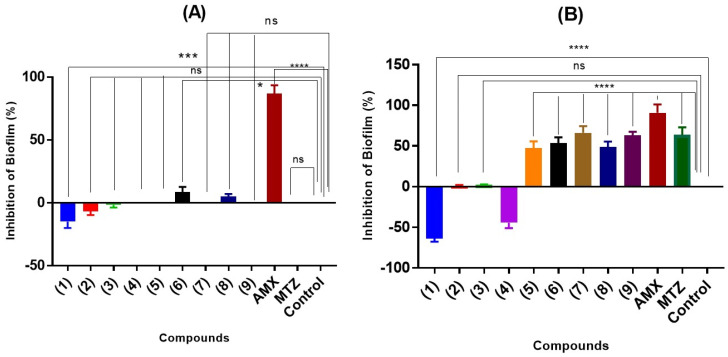
Anti-biofilm (crystal violet assay) activity of thiol and phenol adducts (**1**–**9**) at 500 μg/mL on *L. fermetum* UCO 979C (**A**) and *L. monocytogenes* 19115 (**B**). The % of biofilm inhibition was evaluated after 12 h of incubation at 37 °C. Data are presented as mean ± SD. The asterisks indicate a significant difference from the control (untreated) with * indicating *p* < 0.05, *** *p* < 0.0005, and **** indicating *p* < 0.0001. ns, not significant.

The effect of the adducts on biofilm formation was evaluated using a crystal violet assay. For this, two bacterial strains capable of forming these structures were chosen: *L. fermentum* UCO 979C [54] and *L. monocytogenes* ATCC 19115 [55]. In parallel, and on the same plate, the antibacterial capacity of the adducts was evaluated again, but this time with resazurin, a method that allows for a clear visual analysis of the effects on bacterial viability. The results of both assays at a concentration of 500 µg/mL on the above-mentioned strains are shown below in Figure 8 for phenolic and thiol adducts, respectively. Semisynthetic adducts epicatechin–phloroglucinol (**1**); epicatechin–pyrogallol (**2**), and epicatechin–resorcinol (**3**) did not have a statistically significant inhibitory effect on the biofilms formed. Moreover, adducts (**1**–**3**) had the potential to promote biofilm formation. This enhancing activity is significant in the case of epicatechin–phloroglucinol (**1**) over *L. fermentum* (20%) and *L. monocytogenes* (68%). The same result is observed in the case of thiol adduct (4), with a biofilm-promoting activity of around 44%. As expected, the biomass of the quantified biofilm was decreased in the presence of the corresponding antibiotic to which the strain is sensitive. As seen in Figure 8, the formation of *L. fermentum* UCO 979C biofilm is practically not inhibited by the presence of thiol adducts (**4**–**9**). Only the control with 100 μg/mL of amoxicillin is able to inhibit biofilm synthesis by approximately 80%. Compounds (**6**) and (**8**) have a weak activity on this strain with an inhibition of 8% and 5%, respectively. In the case of *L. monocytogenes*, the adducts generally present a greater inhibition in the formation of biofilms. Adducts (**7**) and (**9**) at 500 µg/mL inhibit formation by 67% and 62%, respectively. However, although adducts (**5**), (**6**), and (**8**) at this concentration do not achieve 50% inhibition, their percentages cannot be considered negligible.

It should be noted that the use of *L. fermentum* as a non-pathogenic control strain allowed us to see that none of the synthesized adducts can inhibit the formation of its biofilm. This result is positive given the large number of probiotic functions that this microorganism provides when it is in its sessile form. It has been shown that the presence of probiotic strains such as *L. fermentum* in the intestinal or oral mucosa has beneficial effects on an organism since they produce compounds such as bacteriocins and biosurfactants, which inhibit the adhesion and growth of intestinal or urogenital pathogenic strains [56]. This agrees with the increase in the biomass of LcY biofilms *Lactobacillus casei* from the fermented milk-based drink Yakult^®^ (LcY), cultured in the presence of EGCG [57]. This could be explained by previous findings indicating that polyphenols can behave as prebiotics due to their modulating effect on the intestinal microbiota [58,59], especially their stimulation of beneficial bacteria such as *Lactobacillus* sp. and inhibition of pathogenic bacteria such as *Clostridium* spp. [60]. Although one can speculate on the stimulant effect of biofilms shown by compounds such as (**1**) and (**4**) included in the present work, some mechanisms have been proposed. For instance, certain antibiotics could increase the production of biofilms at subinhibitory concentrations through the modulation of biochemical pathways that trigger quorum sensing signals, increase EPS synthesis, or increase the levels of second messengers like c–di–GMP and c-di–AMP [61]. So, compounds like 2–hydroxy–1,4-naphthoquinone, riboflavin, and 9,10-anthraquinone–2–sulfonic acid promote biofilm synthesis via modulation of extracellular electron transfer (EET) [62]. It has been described that the inhibition of the c–di–GMP signaling pathway can decrease the formation of bacterial biofilms and promote their dispersion. Therefore, changes in environmental factors—which may vary with laboratory culture conditions—also affect the cycle of bacterial biofilms, such as the case of low-oxygen environments that can facilitate the dispersion of bacterial biofilms by accelerating the degradation rate of c–di–GMP [63]. On the contrary, antibiotics such as levofloxacin have been reported to increase the intracellular concentration of c–di–GMP, thereby increasing the production of EPS and stimulating the formation of biofilms, thus promoting resistance to antibiotics [64]. In this way, the integration of biological and physical models is the key to understanding the dynamics of bacterial biofilms [65]. In some cases, the stimulation of the formation of certain biofilms can be a desired effect; as mentioned above, in the case of probiotics, the stimulation of biofilms brings beneficial effects to the organism. Similarly, the benefits of bacterial biofilms at various levels have been highlighted. It is known that the presence of plastics and microplastics in various water bodies has harmful effects on ecosystems and the health of the population [66]. The removal of these microplastics is a difficult task due to their very small size, buoyancy, and easy dispersion. However, it has been discovered that these microplastics allow for the formation of biofilms, which increases the coagulation and flocculation of these structures, facilitating their elimination [67]. Consequently, methods have been developed to trap microplastics using biofilms, as in the case of Liu and collaborators, who developed a method to capture and release microplastics as needed using wild-type and modified strains of *P. aeruginosa* [68]. Therefore, the search for new molecules capable of stimulating the formation of biofilms as a method to reduce microplastic pollution is part of the challenge [69]. The results of the resazurin assay are shown below in Table 6. In the case of viable *L. fermentum*, a yellow color is observed instead of the characteristic pink-red color of resorufin. This is because this strain is classified as a “lactic acid bacteria” that is capable of producing lactic acid, acetate, CO_2_, and ethanol, depending on environmental conditions (aerobiosis or anaerobiosis). The presence of lactic acid decreases the pH and induces a change in the color of resazurin. So, below pH 3.8, resazurin is orange-yellow, and above pH 6.5, it is purple [70,71]. Consequently, treatment with the nine adducts does not affect the viability of *L. fermentum*.

Regarding the effect of the thiol adducts upon *L. monocytogenes*, adducts (**5**), (**6**), and (**9**) are the ones that present the best results, standing out at concentrations of 1000 µg/mL. For these compounds it is clear that resazurin does not change color, maintaining the characteristic bluish tone indicative of non-viable bacteria. The results of this assay are in line with those from the diffusion test. Overall, adducts (**5**), (**6**), and (**9**) present the best results in terms of antibacterial activity. As stated in previous paragraphs, this effect is most likely due to the increase in lipophilic character. However, the contribution of aromatic and hydroxyl groups in the structure of epicatechin–2-aminothiophenol (**5**), epicatechin–2–methylthiophenol (**6**), and epicatechin–1,2-ethanedithiol (**9**), cannot be ruled out [72].

## 3. Materials and Methods

### 3.1. Chemicals

Phloroglucinol (≥99.0%), resorcinol (≥99.0%), pyrogallol (≥99.0%), captopril (≥98.0%), 2-mercaptoethanol (≥99.0%), 2-amino-thiophenol (≥98.0%), 2-methyl-thiophenol (≥98.0%), 1,2-ethanedithiol (≥98.0%), and methyl-thioglycolate (≥98.0%), were purchased from Merck (Darmstadt, Germany). Catechin (≥99.0%) and epicatechin (≥97.0%), were purchased from Sigma-Aldrich (St. Louis, MO, USA). Procyanidin B2 was purified from avocado peel extract according to [73]. Acetonitrile, methanol, and acetic acid were HPLC-grade and acquired from Merck. All other reagents and solvents were analytical grade [73].

### 3.2. Avocado Peel Extraction

Avocado peels were removed from avocado fruits (*Persea americana* Mill. var Hass) acquired at a local market in Chillán, Chile. A batch of 1000 g of peels was immediately poured into 5000 mL of water at 80 °C for 1 h with a continuous agitation speed of 150 rpm. After the maceration step, homogenization was performed using a blender (20.000 rpm × 1 min). Aqueous extracts were filtered through a double layer of cheesecloth and loaded on a column (40 × 5 cm) containing Sepabeads SP-850 (Supelco, Bellefonte, PA, USA) stationary phase, pre-conditioned with distilled water [73]. The column was washed with 3 bed volumes of water. Polyphenols were recovered with 100% methanol. The methanol extract was concentrated under vacuum and finally freeze-dried (31 g per on fresh weight basis) and stored at −80 °C until use for the adducts’ semi-synthesis and CPC fractionation.

### 3.3. HPLC and TLC Analysis

The polyphenols present in the avocado peel extract were identified by RP-HPLC according to previous work [59,74,75]. A YL9111S binary pump coupled to a YL9120s UV/Vis detector (Young Lin^®^) was used. The chromatography system was equipped with a 250 × 4.6 mm, 5 μm Kromasil KR100-5C18 column (Eka Chemicals AB, Bohus, Sweden). The solvent system was composed of solvent A (ultrapure water containing 2% acetic acid, *v*/*v*) and solvent B (100% methanol). The following gradient program was used: 0–45 min, 15–80% B; 80–15% B; and 45–46 min. Finally, the column was re-equilibrated for an additional 10 min. The flow rate was 0.8 mL/min. Detection was performed by using UV-VIS chromatograms acquired at 350 nm for flavonols, 320 nm for phenolic acids, 280 nm for flavan-3-ols, and 520 nm for anthocyanins. TLC analysis was performed on TLC silica gel pre-coated 60 F254 plates (Merck) to confirm of the progress of the depolymerization reaction and the formation of the different adducts. The mobile phase was toluene: acetone: formic acid 3:6:1 *v*/*v*/*v*. After TLC development, plates were dried under a nitrogen stream and derivatized with the reagent DMACA [75]. Epicatechin and procyanidin B2 were used as standard.

### 3.4. Semi-Synthesis of Flavan-3-ol Adducts with Phenol and Thiol Nucleophiles

For phenolic adducts, semi-synthesis from PAC adducts was carried out according to the methodology detailed in our previous work [12]. In this protocol, 1 g of avocado PACs was mixed with 4 g of phloroglucinol, resorcinol, or pyrogallol, 3.2 g of ascorbic acid, and 5.6 mL of concentrated HCL in 200 mL methanol. After incubation in a water bath at 35 °C for 20 min with stirring, the reaction was stopped with 100 mL of 400 mM sodium acetate [10]. Due to the high polarity of the phenolic adducts, the ethyl acetate extraction was incomplete. Therefore, the reaction mix was concentrated under vacuum, diluted with water, and adsorbed into an Amberlite XAD-7 HP column (40 × 3.0 cm) pre-conditioned with a water column. The column was rinsed with water until all impurities (ascorbic acid and salts) were removed. Phenolic adducts were recovered with 100% methanol. After concentration under vacuum (<40 °C), the residue was finally freeze-dried. In the case of the thiol adducts, the reaction conditions were similar for the formation of the 6 adducts, following the protocols published previously [24,76]. As thiol adducts are more lipophilic than their phenolic counterparts, three times extraction with ethyl acetate was sufficient to recover all products. Methanol was used as the medium for all reactions except for the captopril adduct, where ethanol was preferred. The medium also was acidified with 37% fuming HCl. The incubation time was different in each case; it was set according to the progress of the reaction (disappearance of PAC oligomers), which was monitored by TLC-DMACA. After the incubation period, the reaction was stopped with distilled water and neutralized by adding a 0.1 M NaHCO_3_ solution until reaching pH 7.0. 

### 3.5. HPLC-ESI-QTOF-MS-MS

Semi-synthetic compounds were analyzed using an HPLC system coupled in series to a diode array detector (DAD) and a triple quadrupole mass spectrometer. The overall system sequence was as follows: SIL-30 AC Nexera Autosampler, a Nexera LC-30 AD Liquid Chromatograph, CTO 20 AC column oven, SPD M 20 A Prominence DAD, and a CBM 20A communication bus (all from Shimadzu, Kyoto, Japan). Mass spectra were acquired using a QTrap 3200 LC/MS/MS system (Applied Biosystems/MDS Sciex, Framingham, MA, USA). A C-18 solid core particle column (150 × 4.6 mm i.d. with 2.7 μm particles; Halo, Advanced Materials Technology, Inc., Wilmington, DE, USA) was used. The mobile phase was composed of A (acetonitrile) and B (0.05% aqueous formic acid, *v*/*v*) with a linear gradient elution: 0–16 min, 55% A; 16–36 min, 55–65% A; 36–46 min, 65–77% A; 46–56 min, 77–80% A; and 56–70 min, 80–95% A. Re-equilibration was 20 min between individual runs. The mobile phase flow rate was 0.4 mL/min, and the column temperature was maintained at 35 °C. Detection using DAD was performed at 280 nm. The proposed identities are based on the mass spectra of the analytes with those of the reference compounds when these compounds were available and by comparison with the literature data. The mass spectrometer was used with electrospray ionization (ESI) and was operated in negative mode. The source temperature was set at 450 °C, the nebulizer gas pressure at 2.7 bar, and the auxiliary gas pressure at 3.4 bar. The *m*/*z* mass range was set to 100–1200. For the analysis of the compounds at high resolution, an HPLC-DAD-QTOF-MS/MS Compact system (Bruker Daltonics GmbH, Bremen, Germany) was used. Instrument control and data collection were carried out using Compass DataAnalysis 4.4 SR1 (Bruker Daltonics GmbH). The mass spectrometer was operated in positive and negative ESI acquisition modes, over a mass range of 50 to 1500 *m*/*z*, with a scan duration of 0.2 s, and the data were collected in centroid mode. The collision energy was set to a variable range between 10 and 25 eV in stepwise mode. The source parameters were set as follows: end plate offset of 500 V; capillary voltage of 3500 V for −ESI and 4500 V for +ESI; nebulizer pressure of 4 bar; dry gas flow of 9 L/min; and dry temperature of 200 °C. The conditions for auto MS/MS were set as follows: four precursors/cycle and active exclusion after a spectrum. Internal calibration was performed with sodium formate (10% formic acid, 1 M) with mass precision < 3 ppm. MS detection was performed using a base peak chromatogram (BPC). Identity assignment was performed using the PUBCHEM, METFRAG, METLIN, HMDB, and KEEG databases, according to a previously published definition [77]. MS data were also compared with those from other publications.

### 3.6. CPC Separation Procedure

For the isolation of flavan-3-ols adducts from avocado peel PACs, a CPC 250-L centrifugal partition chromatograph (Gilson, Villiers-le-Bel, France) with a 250 mL total cell volume was used. The system has a four-way switching valve for operation in either descending or ascending mode. The CPC unit was controlled with a PLC-2050 system (Gilson, France), with an integrated UV detector and fraction collector. CPC separations were performed with a two-phase solvent system composed of hexane–ethyl acetate–methanol–water (HEMWat) with different volume ratios. The solvent mixture was automatically generated by the PLC-2050 equipment. The CPC rotor was first filled with 1.5 column volumes using the lower phase at 30 mL/min and 500 rpm rotation. The upper phase was pumped into the system in ascending mode at a flow rate of 8 mL/min (CPC), and the rotational speed was increased from 0 to 1800 rpm for the adduct purification. After equilibrium was reached, a 2 g sample of the adducts was dissolved in 10 mL 1:1 mixture of the upper and lower layers and injected into the CPC systems through the automatic port. Elution was monitored using scan 200–600; 280, 330, 360, and 430 nm wavelengths, collecting fractions in 25 mL tubes. Fractions with similar composition were reunited according to on-line UV spectra and HPLC and TLC-DMACA profiles (see method in Section 3.4).

#### KD Calculations

The partition coefficients (KD) were calculated according to Ito and coworkers [22], with slight modifications. In brief, after incubation, 100 µL of the reaction mixture (avocado PACs plus nucleophile), were aliquoted in glass vials and evaporated under vacuum in Centrivap. To the dried residues, a 1:1 mix of the corresponding upper and lower phases were added and thoroughly equilibrated by vortexing for 1 min. After complete phase separation, the upper and lower phases were analyzed by HPLC following the method described in Section 3.4. Seven different solvent system were tested (Table 2). Based on the ratio of HPLC peak area of each target compound in the lower and upper phases, the KD values were calculated as follows:

(1) $$\mathrm{KD}=\frac{\mathrm{HPLC}\;\mathrm{peak}\;\mathrm{area}\;\mathrm{of}\;\mathrm{target}\;\mathrm{adduct}\;\mathrm{in}\;\mathrm{upper}\;\mathrm{CPC}\;\mathrm{phase}}{\mathrm{HPLC}\;\mathrm{peak}\;\mathrm{area}\;\mathrm{of}\;\mathrm{target}\;\mathrm{adduct}\;\mathrm{in}\;\mathrm{lower}\;\mathrm{CPC}\;\mathrm{phase}}$$

### 3.7. DPPH Free Radical Scavenging Activity

The free radical scavenging activity of the adducts was assessed by the DPPH assay. This assay is based on the reduction of the stable, purple-colored DPPH^•^ radical to its yellow DPPH_2_ form. This reduction is kinetically monitored at 517 nm for 30 min [78]. The analyses were performed in a 96-well plate format using a freshly prepared DPPH solution (100 μmol/L) in methanol daily. A 200 μL aliquot of this solution was added to the wells (except in the blank wells). Afterward, 25 μL of adducts, samples, controls, or standard were added to each well and mixed. The absorbances were recorded in an EPOCH microplate reader (BioTek Instruments, Winooski, VT, USA). The data were analyzed with the Gen5 software package version 1.11 The adduct concentrations ranged from 7.5 to 45.0 µM in methanol. All determinations were performed in triplicates. The percentage of scavenging was calculated as:

(2) $$\mathrm{Scavenging}\;\mathrm{DPPH}\;\mathrm{rate}=\frac{1-(A1-A2)}{A0}\times 100\%$$

where A0 is the absorbance of the control (without sample), A1 is the absorbance in the presence of the sample, and A2 is the absorbance of the sample without a DPPH radical. The free radical scavenging ability of the samples was expressed as the IC_50_ value, which is the inhibitory concentration at which 50% of the DPPH radical was scavenged. The IC_50_ values were calculated from the area under the curve (AUC) of scavenging activities (%) vs. the logarithm of the concentrations of respective adducts. Epicatechin, epigallocatechin gallate (EGCG), and Trolox were used as controls.

### 3.8. Microbial Culture and Maintenance

The probiotic strains *Limosilactobacillus fermentum* UCO-979C and *Lacticaseibacillus rhamnosus* UCO-25A were activated from strains (Glycerol 20% *v*/*v*, cryopreserved at −80 °C; Bacterial Pathogenicity Laboratory) and cultured under microaerobiosis conditions (10% CO_2_), in Mann–Rogosa Sharpe broth (MRS; BD©, Franklin Lakes, NJ, USA) at 37 °C for 24 h, followed by cultivation on MRS agar (BD©) under the same conditions [54]. The aerobic pathogenic strains *Listeria monocytogenes* ATCC 19115 and *Escherichia coli* ATCC 25922 were activated from strains (Glycerol 20% *v*/*v*, cryopreserved at −80 °C; Bacterial Pathogenicity Laboratory) and were grown under aerobic conditions in trypticase soy broth (TSA; BD©) at 37 °C for 24 h, followed by cultivation on PALCAM agar (Merck©) and TSA (BD©), respectively, under the same conditions [79]. *Staphylococcus aureus* ATCC 9144, *Escherichia coli* (Migula) ATCC 11775, and *Salmonella enterica* ATCC 13076 were obtained from the Cell Culture Laboratory of the Department of Basic Sciences, Universidad del Bío-Bío, Chile. Mueller Hinton Agar (Oxoid^®^, Basingstoke, UK) was used as a plate culture medium and Trypticase Soy culture broth (Liofilchem^®^, Roseto degli Abruzzi, Italy) plus Yeast Extract (Merck^®^) was used for the bacterial inoculum. These last strains were maintained in trypticase soy broth (TSB) medium containing 50% (*v*/*v*) glycerol at −80 °C.

#### 3.8.1. Screening Disk and Well-Diffusion Test Susceptibility

The antibacterial potential of different adducts was analyzed in terms of the zone of inhibition. In the case of phenolic adducts, the well-diffusion method was used. The plate diffusion test was performed to approximate the antibacterial power of the adducts. Standard disks with currently marketed medications (gentamicin and chloramphenicol) were used to compare these capacities. Gram-(−) and Gram-(+) strains were used to broaden the possible spectrum of action of the molecules. This type of assay provides information not only on the antibacterial capabilities of the adducts but also allows us to understand which structural changes demonstrate better structure–activity results [80]. It was carried out primarily to analyze the susceptibility of the probiotic and pathogenic strains to the compounds tested. We used 100 mm and 25 mL plates of MRS, PALCAM, and Müeller-Hinton (BD©) agar, to which 7 to 9 wells (6 mm in diameter) were made equidistant with a sterile Pasteur pipette. Bacterial suspensions were prepared in sterile saline adjusted to McFarland 0.5 for the strains *L. fermentum* UCO 979C, *L. rhamnosus* UCO 25A, *L. monocytogenes* 19115, *E. coli* 25922, *E. coli* ATCC 11775, *S. aureus* ATCC 9144, and *S. enterica* ATCC 13076, and were spread using a sterile swab on MRS agar plates, PALCAM and Müeller–Hinton, accordingly. Subsequently, the wells were filled with 50 μL aliquots of the compounds on the inoculated agar surfaces. Since thiol adducts are poorly soluble in water, the Kirby–Bauer disk diffusion test was performed. The plates were incubated for 24 h for probiotic and aerobic pathogenic strains at 37 °C under aerobiosis/microaerobiosis conditions, as appropriate [81]. Antibacterial activity was tested at a single concentration of 500 μg/mL for each compound. In the well-diffusion assay, amoxicillin (AMX) and metronidazole (MTZ) (Sigma-Aldrich^®^) were used as antibiotic control at a concentration of 100 μg/mL, and DMSO 20% and sterile distilled water were used as negative controls. For the thiol adducts, control antibiotic susceptibility disks of gentamicin 10 μg, chloramphenicol 30 μg, and amoxicillin 30 μg were used. All tests were performed in triplicate, and the observed antibacterial activity was expressed as the average of the inhibition diameters (mm) produced by the tested samples.

#### 3.8.2. Biofilm Test on Biofilm Formation

To analyze biofilm formation, 96-well plates with bacterial inoculum in the absence or presence of the compounds and controls were used. Dilutions of the semisynthetic adducts of epicatechin were prepared at a concentration of 5.000 µg/mL, while the antibiotic controls were diluted to 1.000 µg/mL. The bacterial inoculum was prepared in sterile broth at McFarland 0.5 for the probiotic strains and aerobic pathogenic strains. *L. fermentum* UCO 979C was cultured in MRS broth + 2% glucose, while *L. monocytogenes* 19115 was cultured in BHI broth. The distribution in the 96-well plate was as follows: To row A (negative control), containing broth only (3 wells each strain toward the right columns 1–3; 4–6; and 7–9); to Row B broth (control without compounds) plus bacterial inoculum (3 wells each strain toward the right columns 1–3; 4–6; and 7–9). To rows C, D, and E bacterial inoculum was added, and subsequently, DMSO and control antibiotics were added, to obtain concentrations of 2% DMSO and 100 µg/mL of antibiotics, respectively, in each well. Finally, in Row F, the compounds (1–9) at 500 µg/mL (final concentration) were tested on strains dispersed in broth [82]. The plates were incubated at 37 °C for 12 h for strains *L. fermentum* UCO 979C and *L. monocytogenes* 19115. After incubation, using an 8-channel multichannel micropipette by aspiration, 100 µL of culture medium and unbound cells were removed and allowed to dry for 10 min. Subsequently, the biofilms obtained were washed 3 times with 200 µL of phosphate buffer saline (PBS, Sigma-Aldrich^®^) to eliminate unattached cells and allowed to dry for 10 min. The adhered viable biomass was quantified using the crystal violet assay (CV, Merck©), for which 200 µL of 0.1% CV was added for 10 min, which was subsequently discarded by aspiration and allowed to dry for 10 min. To eliminate excess CV, the stained biofilms were washed 4 times with 200 µL of PBS and allowed to dry for 10 min. Finally, 200 µL of ethanol: acetone mixture (80:20) was added for 10 min, and the optical density (OD) was read at 590 nm in an Infinite^®^ M200 Pro microplate reader (TECAN^®^, Kawasaki, Japan) using i-control 1.9 software for Infinite Reader [4,56]. In parallel to the CV assay, the viability of the biofilm formed was analyzed by adding 0.02% resazurin (RSZ) dissolved in PBS. To perform this step, after washing the wells 3 times with PBS, 100 μL of corresponding sterile broth and 25 μL of 0.02% resazurin were added to the biofilms and incubated at 37 °C under aerobiosis/microaerobiosis conditions for 1–2 h. If the well changed color from blue (resazurin) to pink (resorufin), it meant that the bacteria were still viable [83]. All assays were performed in triplicate in three independent assays.

## 4. Statistical Analysis

The data were entered into Excel and processed using GraphPad Prism Software version 8.0.1 for statistical analyses. Two-way ANOVA with Dunnett’s method of multiple comparisons was used, and a significant difference was considered when *p* < 0.05. The reported values correspond to the arithmetic mean of the determinations and their standard deviation (SD).

## 5. Conclusions

In this work, phenol and thiol nucleophiles were used to obtain nine compounds through semi-synthesis from avocado peel PAC extract. The preparative CPC methodology was successfully used to separate phenolic- and thiol-derived adducts using solvent systems based on hexane–ethyl acetate–methanol–water, allowing for the neat separation of all target compounds in a single run. Overall, the thiol-derived compounds showed concentration-dependent radical scavenging activity (DPPH). Interestingly, the thiol-adducts, such as (**4**) and (**6**–**8**), displayed an antioxidant profile similar to Trolox. Although the phenol-derived adducts did not show antibacterial activity against the Gram-positive or Gram-negative strains, thiol-derived adducts **4**–**9** showed a more selective activity on pathogenic bacteria than on probiotic strains. The semisynthetic adducts did not have a statistically significant inhibitory effect on the formed biofilms. However, adducts have the potential to promote biofilm formation, with epicatechin–phloroglucinol (**1**) having a significant effect over *L. fermentum* (20%) and *L. monocytogenes* (68%). Thiol adducts (**5**), (**6**), and (**9**) exhibited the best antibacterial activity against *L. monocytogenes* at concentrations of 1000 µg/mL. The improved effect of such compounds is likely due to an increased lipophilic character, but the contribution of aromatic and hydroxyl groups should also be considered.

## Figures and Tables

**Table 1:** The solvent system used in CPC and KD values of the target compounds.

Adduct	System	n-Hexane	EtOAc	MeOH	H_2_O	Mode	Partition Coefficient (KD) ^1^
1	C	1	9	1	9	Ascending	1.25
2	C	1	9	1	9	Ascending	1.11
3	K	1	2	1	1	Ascending	0.88
4	K	1	2	1	1	Ascending	2.61
5	K	1	2	1	2	Ascending	3.01
6	K	1	2	1	2	Ascending	4.06
7	H	1	3	1	3	Ascending	2.30
8	K ^2^	0.5	1.5	0.5	1.2	Descending	1.92
9	K	1	2	1	2	Ascending	1.01

^1^ KD obtained from the analysis of targeted compound (adducts) in the upper and lower phases by C-18-HPLC. ^2^ Solvent K was modified as reported by Tian et al. 2020 [31].

**Table 2:** Yields of semi-synthetic adducts obtained from avocado PACs.

Adduct	Avocado PACs (g)	Adduct Formed (mg)	Yield * (%)	Purity (%)
1	1	218	21.8	98.7
2	1	222	22.2	98.2
3	1	180	18.0	96.1
4	3	450	15.0	95.4
5	2	440	22.0	94.0
6	1	221	22.1	97.3
7	1	179	17.9	93.5
8	2	313	15.7	97.2
9	0.7	99	14.1	98.1

* Yields were calculated using the dry weight of avocado PACs after purification by CPC (see Section 2.2).

**Table 3:** Antioxidant activity expressed as IC_50_ from the adduct samples.

Compound	IC_50_ μM
**1**	14.01 ± 2.27
**2**	22.12 ± 1.18
**3**	30.08 ± 3.01
**4**	25.32 ± 1.16
**5**	30.16 ± 2.02
**6**	13.11 ± 1.27
**7**	13.41 ± 1.14
**8**	25.51 ± 2.01
**9**	30.13 ± 2.17
Epicatechin	24.01 ± 2.02
EGCG	0.40 ± 1.01
Trolox	18.08 ± 3.52

All results are expressed as mean ± SD from three experiments (*n* = 3).

**Table 4:** Results of antimicrobial susceptibility tests for phenolic adducts (**1**–**3**).

Strain	Inhibition Zone (Mean ± SD, mm)				
	(1) ^2^	(2) ^2^	(3) ^2^	AMX ^1^	MTZ ^1^
*L. fermentum* UCO 979C	(-)	(-)	(-)	23.7 ± 4.7	7.3 ± 1.2
*L. rhamnosus* UCO 25A	(-)	(-)	(-)	20.7 ± 0.6	7.3 ± 1.2
*L. monocytogenes* 19115	(-)	(-)	(-)	26.3 ± 1.5	8.3 ± 1.0
*L. monocytogenes* 7644	(-)	(-)	(-)	15.0 ± 1.0	7.7 ± 0.8
*E. coli* 25922	(-)	(-)	(-)	(-)	(-)
*S. aureus* 9144	(-)	(-)	(-)	(-)	(-)

^1^ The analyzed concentration for AMX and MTZ was 100 μg/mL (5 μg/well). ^2^ Adducts were tested at 500 μg/mL. (-) No activity was observed.

**Table 5:** Results of antimicrobial susceptibility tests for thiol adducts (**4**–**9**).

Strain	Inhibition Zone (Mean ± SD, mm) ^1,2^									
	(4)	(5)	(6)	(7)	(8)	(9)	AMX	MTZ	GEN	CFN
*L. fermentum* UCO 979C	(-)	(-)	(-)	(-)	(-)	(-)	11.3 ± 0.6	7.3 ±1.2	7.3 ± 1.0	Nd
*L. rhamnosus* UCO 25A	(-)	(-)	(-)	(-)	(-)	(-)	9.7 ± 0.6	7.3 ±1.2	7.3 ± 1.2	Nd
*L. monocytogenes* 19115	15.7 ± 1.0	29.1 ± 0.8	16.0 ± 0.2	8.6 ± 0.4	(-)	22.1 ± 0.7	18.6 ± 0.8	7.7 ± 0.4	31 ± 0.6	30 ± 0.6
*L. monocytogenes* 7644	15.0 ± 0.5	30.1 ± 0.8	16.1 ± 0.6	7.2 ± 0.4	(-)	19.2 ± 0.8	15.1 ± 0.8	8.3 ± 0.8	35 ± 0.6	34 ± 0.6
*S. aureus* 9144	18.0 ± 0.6	17.1 ± 0.6	21.2 ± 0.4	(-)	14.1 ± 0.6	19.2 ± 1	27.8 ± 0.6	Nd	28 ± 0.6	35 ± 0.7
*E. coli* 25922	10.2 ± 0.6	15.8 ± 0.6	14.7 ± 0.6	9.9 ± 0.6	8.6 ± 0.6	12.0 ± 0.6	(-)	Nd	22 ± 0.5	32 ± 0.6
*E. coli* 11775	9.1 ± 0.6	17.3 ± 0.3	16.0 ± 0.6	11.1 ± 0.2	9.3 ± 0.6	11.4 ± 0.7	27.03 ± 0.6	Nd	25 ± 0.6	30 ± 0.6
*S. enterica* 13076	14.4 ± 0.4	16.4 ± 0.4	17.2 ± 0.2	12.2 ± 0.2	11.0 ± 0.8	12.0 ± 0.6	Nd	Nd	25 ± 0.6	30 ± 0.6

^1^ Disks of gentamicin (GEN) 10 μg, chloramphenicol (CFN) 30 μg, and amoxicillin (AMX) 30 μg were used. MTZ was used in a well at 100 μg/mL (5 μg/well). ^2^ Adducts were tested at 500 μg/mL. (-) No activity was observed. Nd = not determined.

**Table 6:** Effect of phenol and thiol adducts (**1**–**9**) on the viability of biofilm-forming strains of *L. fermentum* and *L. monocytogenes*.

Strain	Viability ^1^										
	(1)	(2)	(3)	(4)	(5)	(6)	(7)	(8)	(9)	AMX ^2^	MTZ ^2^
*L. fermentum* UCO 979C	(++)	(++)	(++)	(++)	(++)	(++)	(++)	(++)	(++)	(++)	(+)
*L. monocytogenes* 19115	(++)	(++)	(++)	(++)	(+)	(−)	(++)	(++)	(+)	(++)	(+)
*L. fermentum* UCO 979C	(++)	(++)	(++)	(++)	(++)	(+)	(++)	(++)	(++)	(+)	(+)
*L. monocytogenes* 19115	(++)	(++)	(++)	(+)	(−)	(−)	(++)	(++)	(−)	(+)	(+)

^1^ Effects of the adducts (500 μg/mL and 1000 μg/mL) on the viability of *L. fermentum* and *L. monocytogenes* by resazurin assay. (++): viable, (+): medium viability, (–): not viable. ^2^ MTZ and AMX were tested at 100 μg/mL.

## Data Availability

The data presented in this study are contained within this article and in the Supplementary Materials.

## Supplementary Materials

The following supporting information can be downloaded at: https://www.mdpi.com/article/10.3390/molecules29122872/s1, Table S1: Identification of targeted adduct compounds by HPLC–ESI–QTOF–MS-MS in negative ion mode; Figure S1: HPLC-MS of adduct (4); Figure S2: HPLC-MS of adduct (5); Figure S3: HPLC-MS of adduct (6); Figure S4: HPLC-MS of adduct (7); Figure S5: HPLC-MS of adduct (8); Figure S6: HPLC-MS of adduct (9); Figure S7: Disk diffusion test susceptibility of adducts (4–9) on *L. monocytogenes* strains; Figure S8: HPLC-UV trace of thiol adducts (4–9).
