# Supplementary material for: Synthesis and Isolation of Phenol- and Thiol-Derived Epicatechin Adducts Prepared from Avocado Peel Procyanidins Using Centrifugal Partition Chromatography and the Evaluation of Their Antimicrobial and Antioxidant Activity
Source: Molecules. 2024 Jun 17;29(12):2872. doi: 10.3390/molecules29122872 (PMC11206461; doi:10.3390/molecules29122872)
Supplement: Supplementary file 1 [file molecules-29-02872-s001.zip › molecules-2763215-supplementary.pdf]

Supplementary Material

Synthesis and Isolation of Phenol- and Thiol-Derived Epicatechin Adducts Prepared from Avocado Peel Procyanidins Using Centrifugal Partition Chromatography and the Evaluation of Their Antimicrobial and Antioxidant Activity

Barbara Berrios-Henríquez [1], Matías Venegas-Toloza [1,2], María Reyes-Fuentes [3], Felipe Zúñiga-Arbalti [4], Luis Bustamante [5], Apolinaria García-Cancino [1], Julio Alarcón-Enos [2] and Edgar Pastene-Navarrete [2,*]

1 Department of Microbiology, Faculty of Biological Sciences, Universidad de Concepción, Víctor Lamas 1290, Concepción 4030000, Chile; barbaraberrios@udec.cl (B.B.-H.); mavenegas2017@udec.cl (M.V.-T.); apgarcia@udec.cl (A.G.-C.)
2 Department of Basic Sciences, Faculty of Sciences, Universidad del Bío-Bío, Avenida Andrés Bello 720, Chillán 3800708, Chile; jualarcon@ubiobio.cl
3 Department of Biochemistry and Molecular Biology, Faculty of Chemical and Pharmaceutical Sciences, Universidad de Chile, Dr. Carlos Lorca Tobar 964, Independencia, Santiago 8380494, Chile; maria.reyes.f@ug.uchile.cl
4 Department of Clinical Biochemistry and Immunology, Faculty of Pharmacy, Universidad de Concepción, Víctor Lamas 1290, Concepción 4030000, Chile; fzuniga@udec.cl
5 Department of Instrumental Analysis, Faculty of Pharmacy, Universidad de Concepción, Víctor Lamas 1290, Concepción 4030000, Chile; lbustamante@udec.cl

* Correspondence: epastene@ubiobio.cl

Table S1. Identification of targeted thiol-derived adducts by HPLC–ESI–QTOF–MS-MS in Negative Ion Mode.

Name	Pseudomolecular ion formula	Retention time (min)	Theorical m/z	[M-H]-	Error (ppm)	Fragments MS/MS
Monomer 4	$C_{18}H_{17}O_8S^-$	11.4	393.0650	393.0658	-2.0	125.0258 (100); 161.0285 (47); 287.0598 (45); 164.0142 (15); 269.0512 (14); 126.0272 (11)
Dimer 4	$C_{33}H_{29}O_{14}S^-$	11.4	681.1284	681.1325	-6.1	125.0266 (100); 287.0568 (97); 285.0394 (17); 161.0305 (14); 243.0629 (13)
Monomer 5	$C_{21}H_{18}NO_6S^-$	11.4	412.0860	412.0849	2.8	125.0241 (100); 161.0238 (30); 165.0182 (26); 412.0849 (18); 124.0219 (14); 289.0704 (14); 137.0239 (14); 287.0542 (13); 164.0102 (11)
Dimer 5	$C_{36}H_{30}NO_{12}S^-$	11.7	700.1494	700.1500	-0.8	125.0236 (100); 287.0556 (78); 161.0233 (37); 288.0569 (10)
Monomer 6	$C_{22}H_{19}O_6S^-$	11.0	411.0908	411.0920	-2.9	125.0254 (100); 287.0557 (58); 161.0283 (29); 201.0586 (18); 164.0117 (16); 411.1101 (14); 123.0284 (14); 173.0624 (13); 215.0706 (10); 167.0340 (10); 133.0307 (10)
Dimer 6	$C_{37}H_{31}O_{12}S^-$	10.0	699.1542	69.,1567	-3.5	125.0244 (100); 287.0568 (65); 161.0253 (28); 288.0595 (11)
Monomer 7	$C_{17}H_{17}O_7S^-$	8.2	365.0700	365.0728	-7.6	125.0255 (100); 161.0261 (60); 287.0584 (33); 269.0457 (13); 165.0208 (13); 137.0262 (10)
Dimer 7	$C_{32}H_{29}O_{13}S^-$	7.8	653,1334	653.1347	-1.9	575.1200 (100); 125.0251 (77); 287.0567 (55); 576.1232 (32); 161.0252 (30); 413.0884 (15); 423.0729 (11); 243.0302 (11); 164.0126 (10)
Monomer 8	$C_{25}H_{28}NO_9S^-$	8.9	518.1490	518.1456	6.5	125.0237 (100); 287.0535 (63); 161.0250 (29); 164.0132 (18); 289.0724 (17); 165.0185 (11)
Dimer 8	$C_{40}H_{40}NO_{15}S^-$	8.7	806.2124	806.2101	2.9	575.1148 (100); 125.0229 (61); 287.0536 (56); 576.1182 (32); 161.0233 (20); 261.0383 (14); 423.0684 (12)
Monomer 9	$C_{17}H_{17}O_6S_2^-$	9.2	381.0472	381.0495	-6.1	125.0258 (100); 161.0252 (41); 287.0587 (33); 164.0119 (17); 121.0279 (14); 269.0480 (10); 169.0001 (10)
Dimer 9	$C_{32}H_{29}O_{12}S_2^-$	8.6	669.1106	669.1149	-6.5	125.0252 (100); 287.0577 (84); 161.0258 (28); 288.0613 (15)

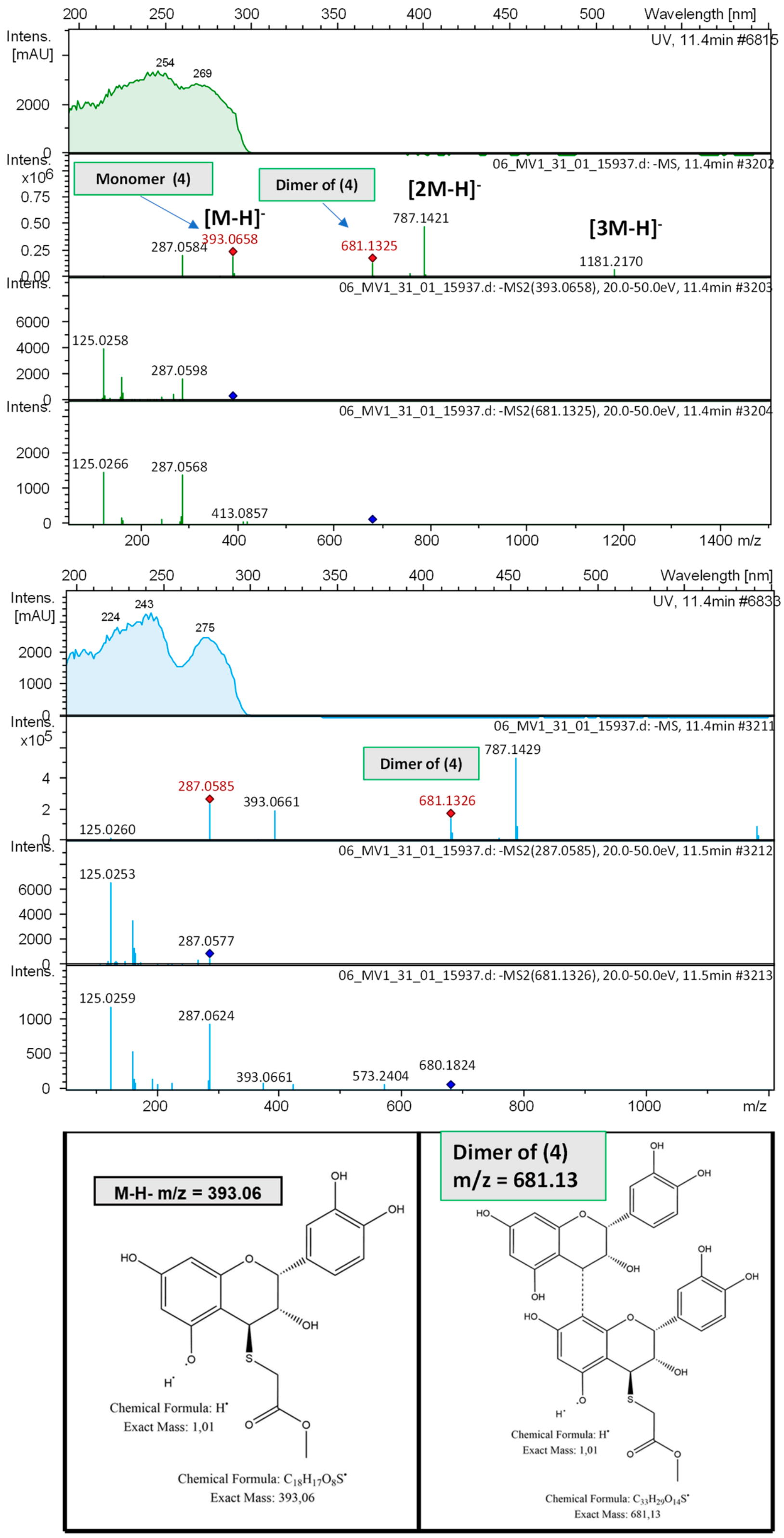

Figure S1. HR-MS of epicatechin thiol-derived adduct (4) and its dimer.

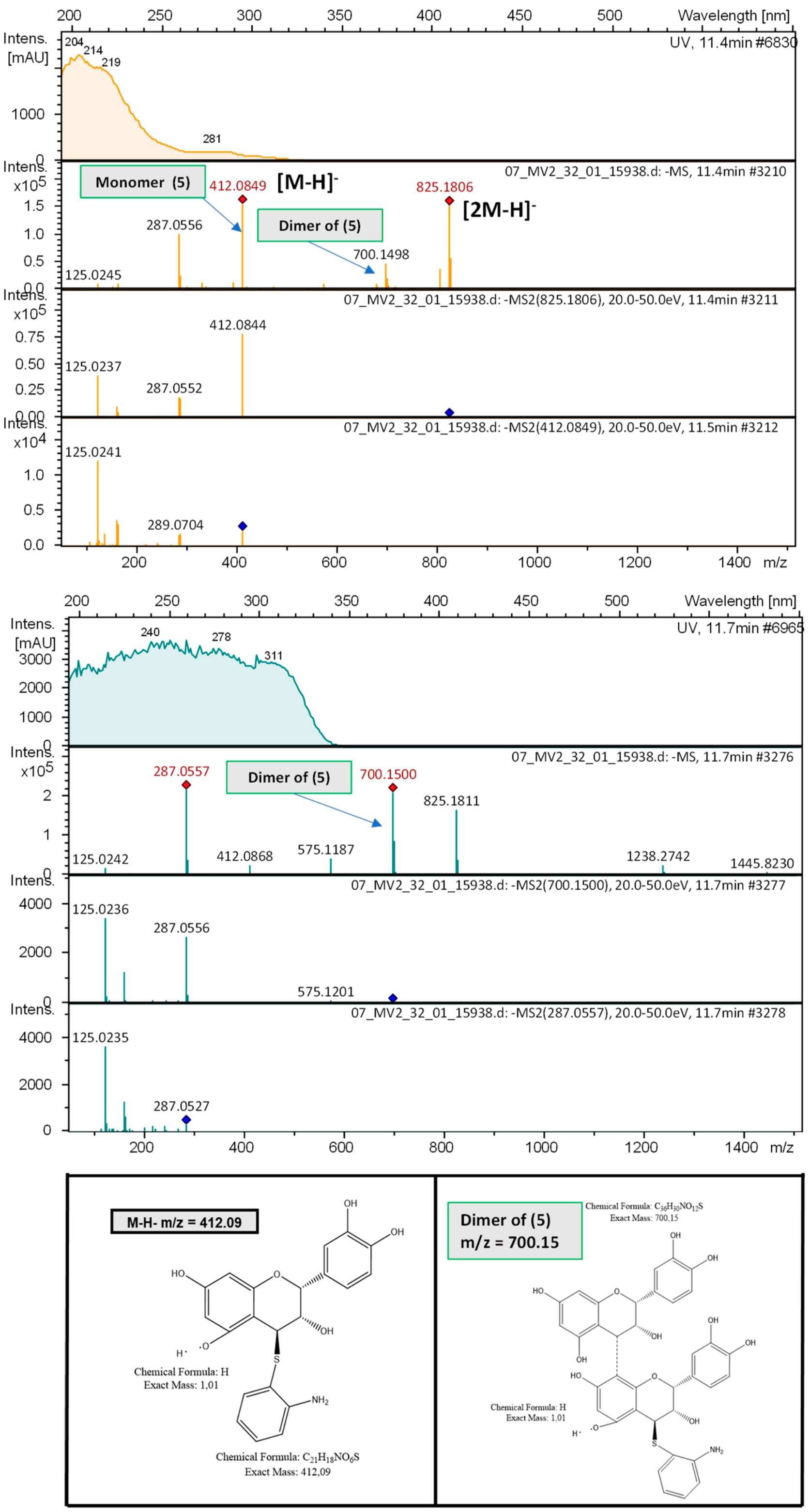

Figure S2. HR-MS of epicatechin thiol-derived adduct (5) and its dimer.

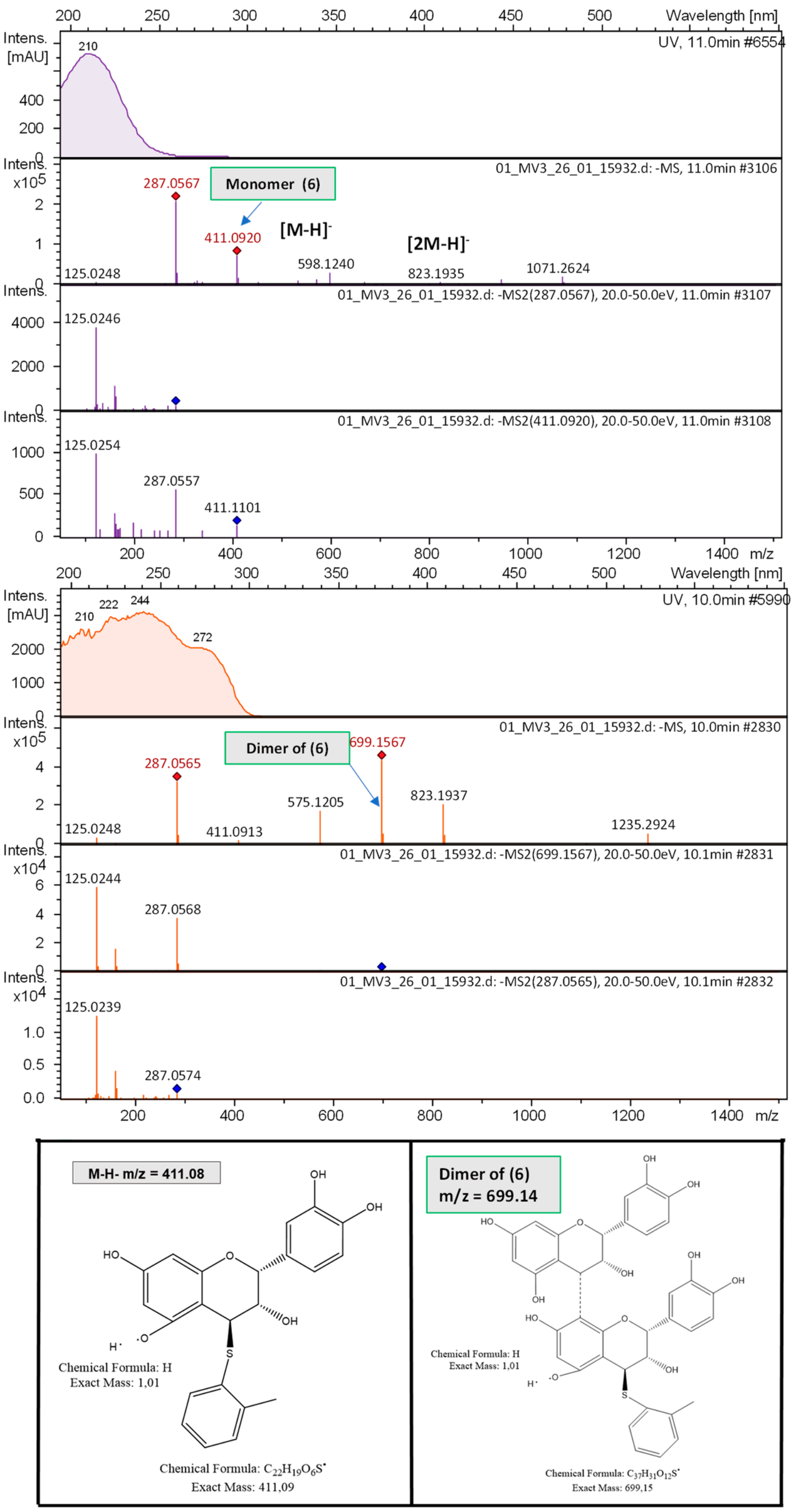

Figure S3. HR-MS of epicatechin thiol-derived adduct (6) and its dimer.

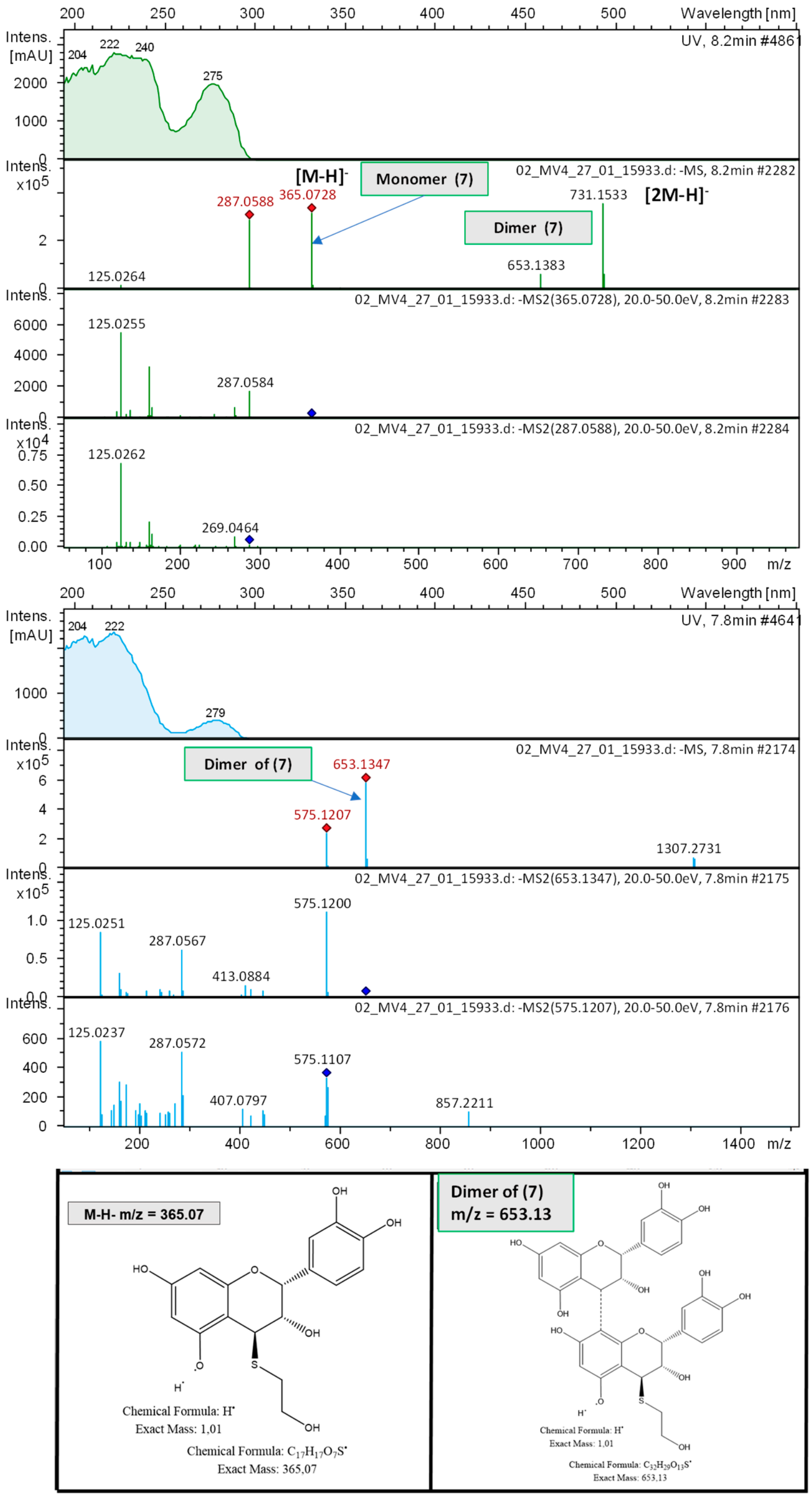

Figure S4 HR-MS of epicatechin thiol-derived adduct (7) and its dimer.

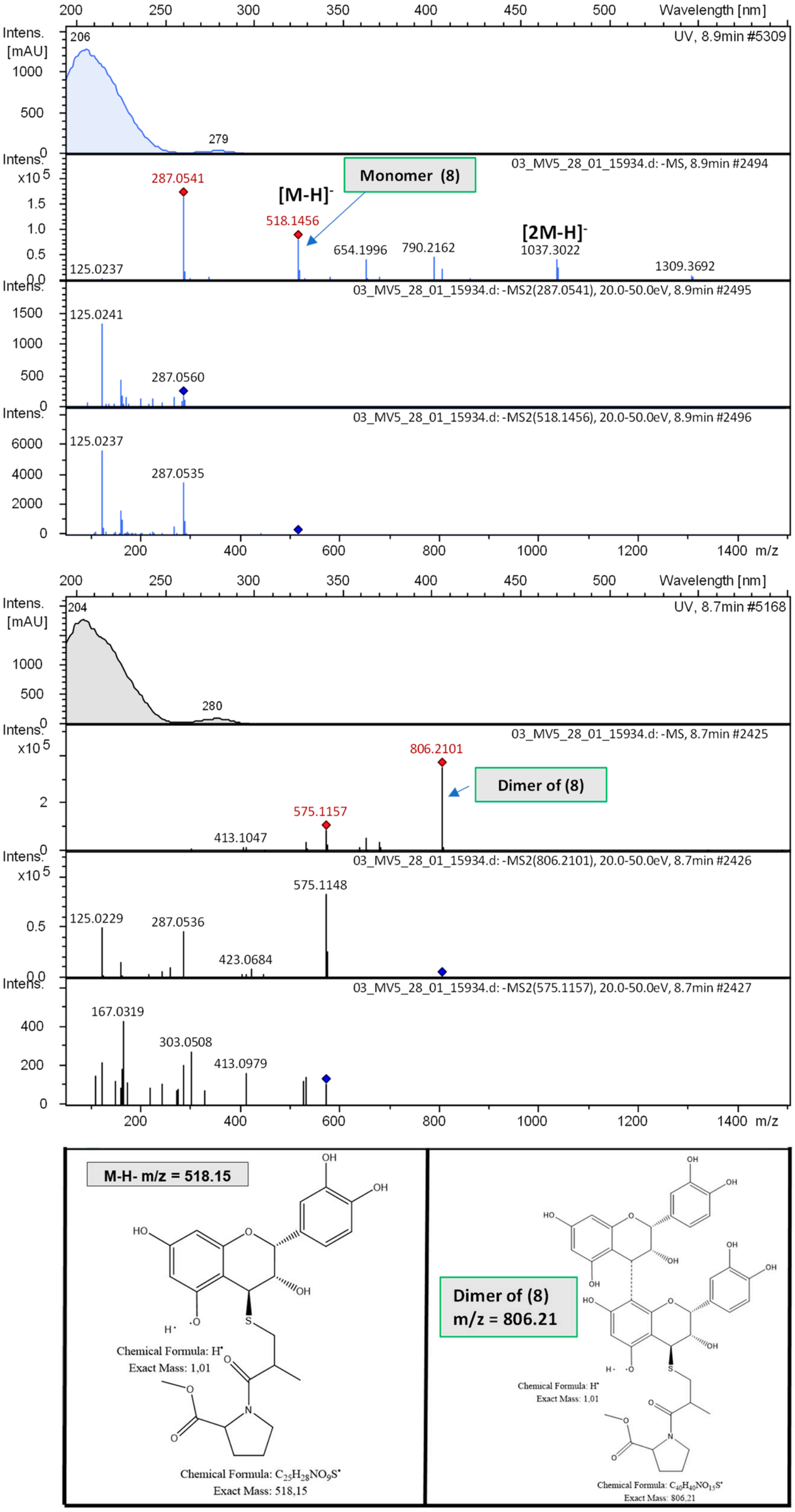

Figure S5. HR-MS of epicatechin thiol-derived adduct (8) and its dimer.

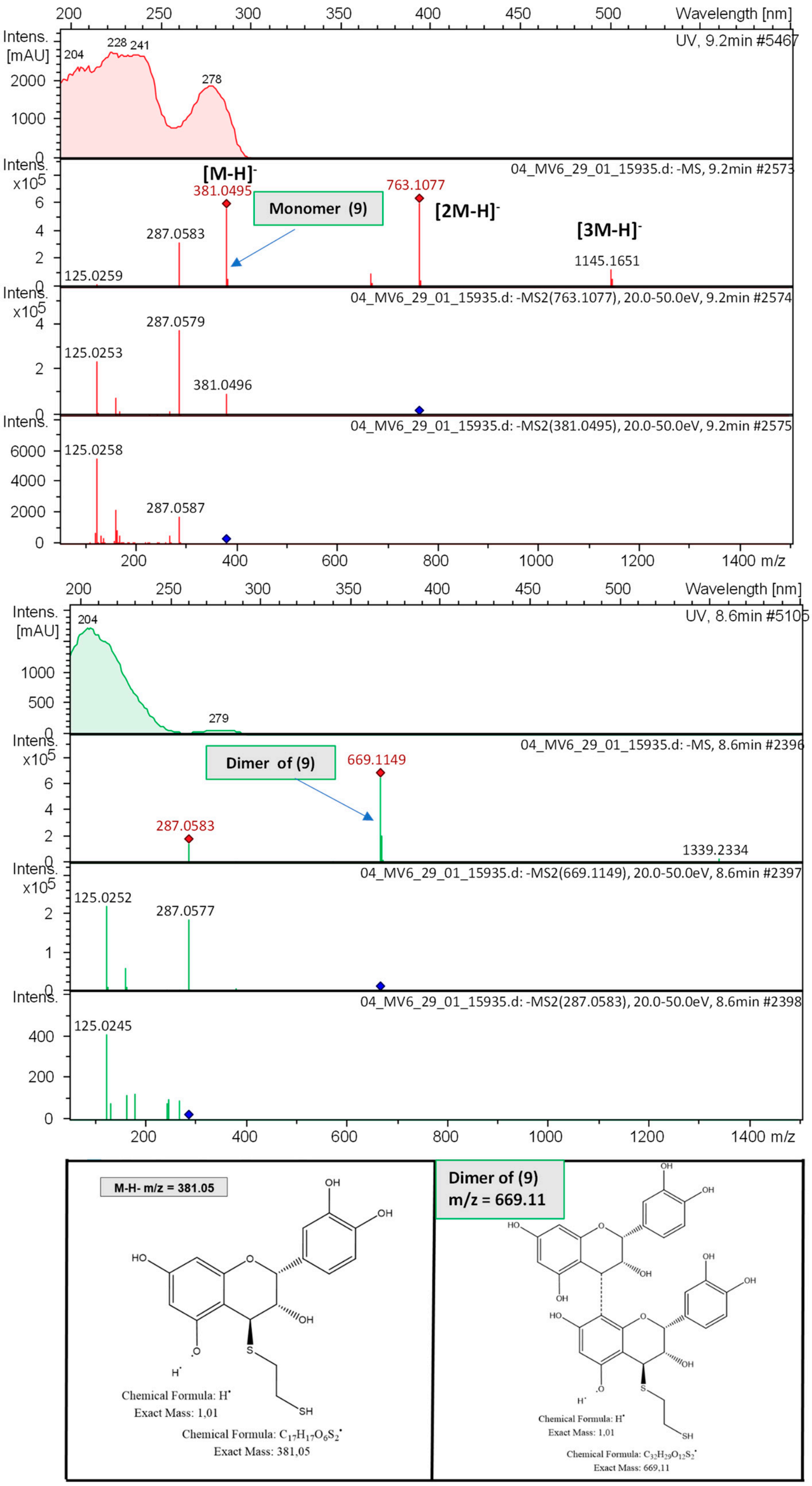

Figure S6. HR-MS of epicatechin thiol-derived adduct (9) and its dimer.

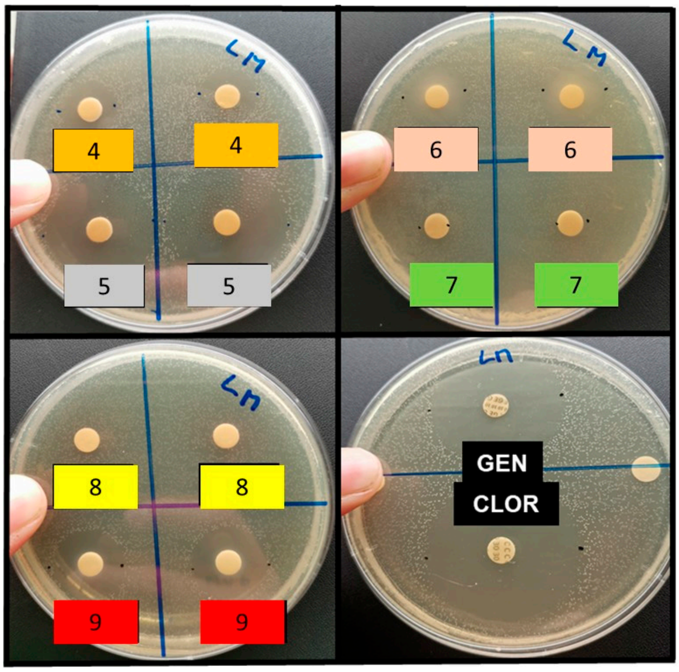

Figure S7. Representative figure of disk diffusion assay for thiol-derived adducts (4-9).

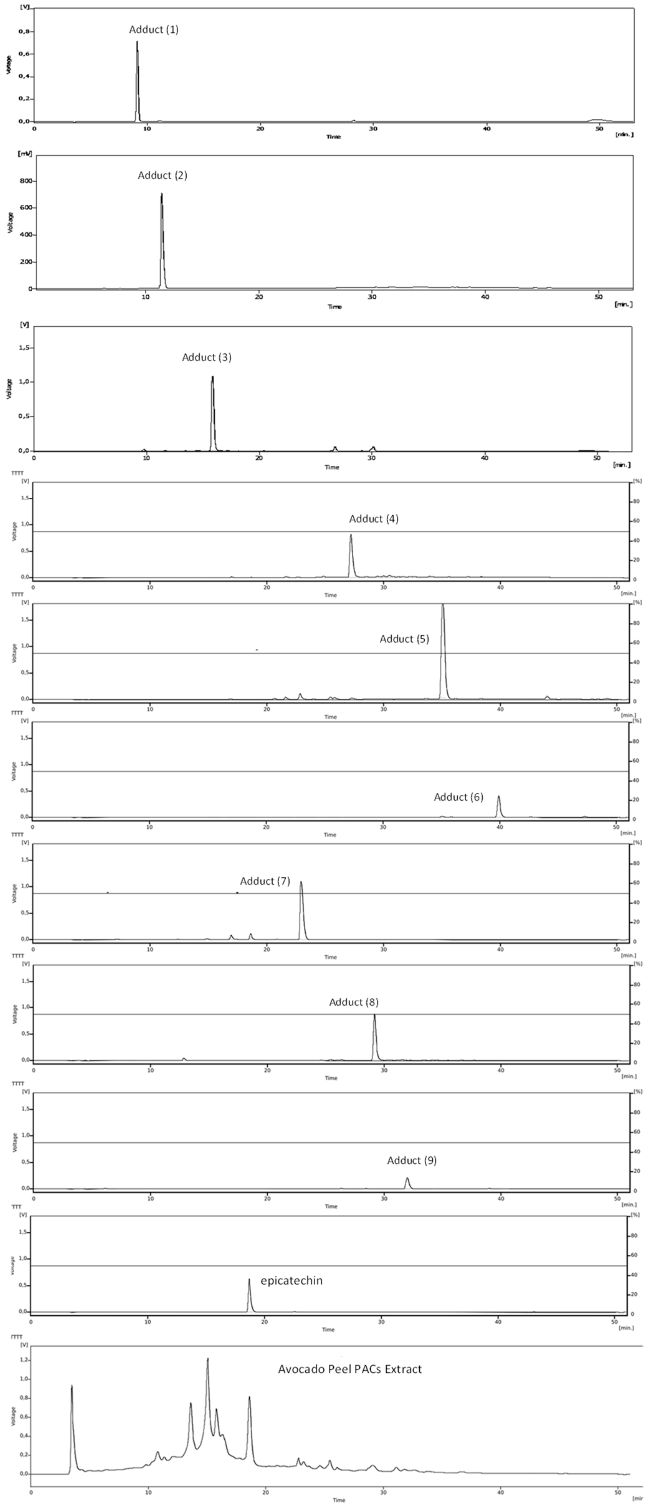

Figure S8. HPLC-UV profiles of phenol and thiol-adducts (1-9), isolated by CPC.
